# Chermebilaenes A and B, New Bioactive Meroterpenoids from Co-Cultures of Marine-Derived Isolates of *Penicillium bilaiae* MA-267 and *Penicillium chermesinum* EN-480

**DOI:** 10.3390/md18070339

**Published:** 2020-06-28

**Authors:** Ling-Hong Meng, Xiao-Ming Li, Hong-Lei Li, Bin-Gui Wang

**Affiliations:** 1 Key Laboratory of Experimental Marine Biology, Institute of Oceanology, Chinese Academy of Sciences, Nanhai Road 7, Qingdao 266071, China; lixmqdio@126.com (X.-M.L.); lihonglei428@126.com (H.-L.L.); 2 Laboratory of Marine Biology and Biotechnology, Qingdao National Laboratory for Marine Science and Technology, Wenhai Road 1, Qingdao 266237, China; 3 Center for Ocean Mega-Science, Chinese Academy of Sciences, Nanhai Road 7, Qingdao 266071, China

**Keywords:** *Penicillium chermesinum*, *Penicillium bilaiae*, co-culture, secondary metabolites, antimicrobial activity

## Abstract

The co-cultivation of two or more different microbial strains in one culture vessel was supposed to be a viable experimental approach for enhancing the diversity of the compounds produced. Two new meroterpenoid derivatives, chermebilaenes A (**1**) and B (**2**), together with three known sesquiterpenoids, sesquicaranoic acid B (**3**), cyclonerodiol (**4**) and bisabol-l-on-13-säuremethylester (**5**), were characterized from a co-culture of the marine-derived fungal isolates of *Penicillium bilaiae* MA-267 and *Penicillium chermesinum* EN-480. Neither fungus produced these compounds when cultured alone under the same conditions. Compound **1** represents an unprecedented acorane-type sesquiterpene hybridized with an octadecadienoic acid skeleton. The structures were elucidated on the basis of spectroscopic analysis, and the absolute configurations were assumed on the basis of acidic hydrolysis combined with modified Mosher’s method and electronic circular dichroism (ECD) calculations. Compound **1** showed potent inhibitory activities against *Ceratobasidium cornigerum* and *Edwardsiella tarda*.

## 1. Introduction

**Figure 1:**
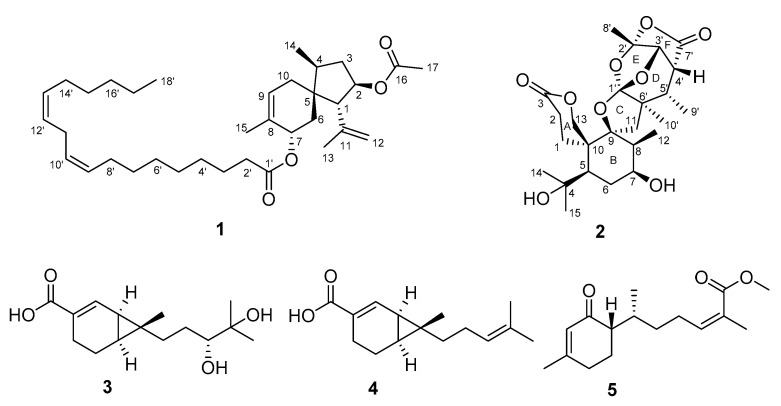
Structures of compounds **1**–**5**.

Marine fungi are able to synthesize a wide range of structurally unique secondary metabolites endowed with numerous biological activities [1,2,3]. However, mining the full-genome sequences of fungi demonstrates their potential to produce many more compounds than previously expected. It is well recognized that most of the microbial biosynthesis gene clusters remain silent and are apparently not transcribed under conventional cultivation conditions [4,5]. The co-cultivation of two or more different microbial strains in one culture vessel was supposed to be a viable experimental approach for enhancing the diversity of the compounds produced [6,7,8]. In our efforts to identify new bioactive secondary metabolites from marine-derived fungi, we previously investigated the secondary metabolites of two strains of the fungal genus *Penicillium*, namely *P. bilaiae* MA-267 and *P. chermesinum* EN-480, from which several sesquiterpenes with a tricyclo (6.3.1.0^1,5^)dodecane skeleton [9], spiromeroterpenoids containing a drimane-type sesquiterpene skeleton [10], and sesquiterpenoids [11] have been isolated, respectively. In order to investigate the chemical potential of *P. bilaiae* MA-267 and *P. chermesinum* EN-480, we initiated the co-culture fermentation of these two fungal strains, which showed the production of several metabolites that were not produced when the two fungi were cultured alone (Figure S23, Supplementary Material). As a result, chermebilaene A (**1**), the first natural sesquiterpene hybridized with octadecadienoic acid, together with a new orthoester meroterpenoid, chermebilaene B (**2**), as well as three known sesquiterpenoids, sesquicaranoic acid B (**3**), cyclonerodiol (**4**) and bisabol-l-on-13-säuremethylester (**5**) (Figure 1), were isolated from the co-culture extract of *P. bilaiae* MA-267 and *P. chermesinum* EN-480. Herein, details of the isolation, structure determination, and biological activities of these compounds are described.

## 2. Results and Discussion

### 2.1. Structure Elucidation of the New Compounds

**Figure 2:**
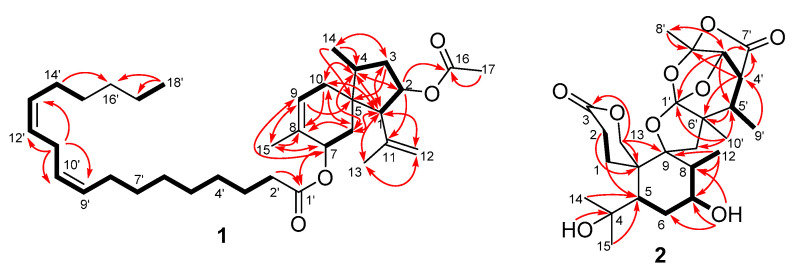
Key COSY (homonuclear chemical shift correlation spectroscopy) (bold line) and HMBC (heteronuclear multiple bond correlation) (arrow) correlations of compounds **1** and **2**.

Chermebilaene A (**1**) was isolated as a colorless oil. Its molecular formula was determined as C_35_H_56_O_4_ by (+)-HRESIMS (high resolution electrospray ionization mass spectroscopy) data (Figure S1, Supplementary Material), implying eight degrees of unsaturation (index of hydrogen deficiency). Analysis of the ^1^H NMR spectrum (Table 1) and HSQC data revealed the presence of five methyl signals at *δ*_H_ 1.76 (H-13, 3H, s), 1.08 (H-14, 3H, d, *J* = 7.1 Hz), 1.63 (H-15, 3H, s), 2.00 (H-17, 3H, s), and 0.89 (H-18’, 3H, t, *J* = 6.8 Hz), five olefinic methines 5.49 (H-9, 1H, dd, *J* = 3.3, 1.8 Hz), 5.40 (H-9’/13’, 2H, dd, *J* = 10.9, 5.5 Hz), 5.34 (H-10’/12’, 2H, dd, *J* = 10.9, 6.5 Hz), and two oxymethines 5.21 (H-2, 1H, td, *J* = 8.6, 4.2 Hz) and 5.38 (H-7, 1H, m), as well as a terminal methylene resonating at *δ*_H_ 4.69 and 4.98 (H-12, 2H, s). In addition, several unresolved methylene signals at *δ*_H_ 1.31, attributable to the protons of a long unbranched carbon chain, were also present. The ^13^C NMR data along with the DEPT (distortionless enhancement by polarization transfer) spectra revealed the presence of 35 carbon atoms, including five nonprotonated carbons (with two olefinic and two carbonyl), nine methines (with five olefinic and two oxygenated aliphatic), sixteen methylenes (with one terminal), and five methyls. Detailed analysis of the NMR data disclosed the structure of **1** to possess an acorane-type sesquiterpenoid residue linked with an octadecadienoic acid through esterification. Specifically, the ^1^H and ^13^C chemical shifts for the left portion of **1** were nearly identical to those of the 9,12-octadecadienoic acid [12], while for the right portion, quite similar to those of the adametacorenol A, an acorane sesquiterpene derivative was obtained from the rice culture broth of a marine-derived fungus, *Penicillium adametzioides* AS-53 [13]. However, signals for the oxygenated methine resonating at *δ*_C/H_ 66.4/3.96 (CH-7) in adametacorenol A [13] were deshielded at *δ*_C/H_ 71.3/5.38 (CH-7) in the NMR spectra of compound **1**, while the chemical shift of carboxyl resonating at *δ*_C_ 178.4 (C-1) in 9,12-octadecadienoic acid [9] shifted upfield at *δ*_C_ 173.8 (C-1’) in **1** due to the esterification effect. In addition, signals for the ortho- and meta-positions resonating at *δ*_C_ 136.6 (C-8) and *δ*_C/H_ 120.9/5.25 (CH-9) in adametacorenol A [13] moved to *δ*_C_ 132.1 (C-8) and *δ*_C/H_ 125.3/5.49 (CH-9) in **1**, respectively. This deduction was further supported by the COSY and HMBC correlations (Figure 2). This is the first time that this type of meroterpenoid, which contains an unprecedented acorane-type sesquiterpene hybridized with octadecadienoic acid skeleton, has been described. 

**Figure 3:**
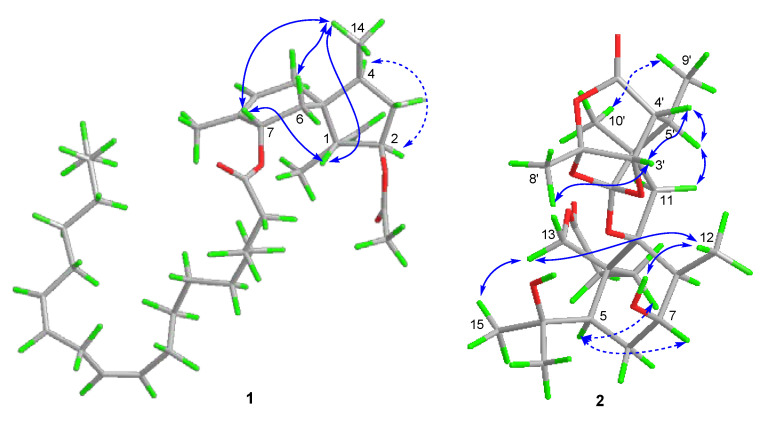
NOESY (nuclear overhauser effect spectroscopy) correlations of compounds **1** and **2**.

The relative configuration of 1 was determined by the analysis of the *J*-values and NOESY (nuclear overhauser effect spectroscopy) data (Figure 3). The coupling constants between H-9’/13’ and H-10’/12’ (10.9 Hz) suggested the geometry of the C=C bond at C-9’ and C-13’ to be Z. Moreover, NOE correlations from H_3_-14 to H-1, H-6*β*, and H-7, and from H-7 to H-1, suggested the same orientation of these groups, while the correlations from H-2 to H-4 placed these protons on the opposite face. 

**Figure 4:**
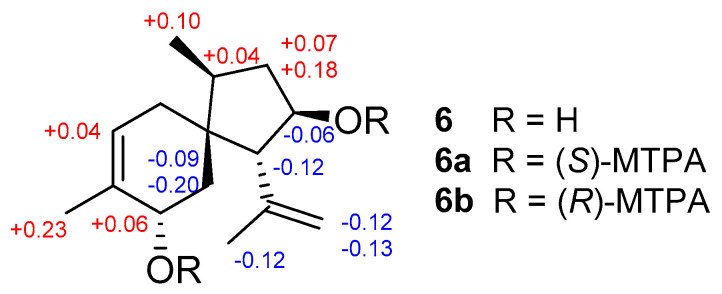
Δ*δ* values (Δ*δ* (in ppm) = *δ_S_ – δ_R_*) obtained for the (*S*)-and (*R*)-MTPA (*α-*methoxy-*α*-(trifluoromethyl) phenylacetyl) esters of compound **6**.

To confirm the structure and absolute configuration of **1**, we pursued a strategy consisting of the acidic hydrolysis of **1** to yield the sesquiterpene diol (**6**) and 9,12-octadecadienoic acid (**7**), followed by modified Mosher’s method for the obtained **6**, and NMR analysis of the resulting Mosher esters allowed the assignment of the 2*R* and 7*S* absolute configuration in compound **6** (Figure 4) and, consequently, the absolute configuration of compound **1** was deduced to be 1*R*, 2*R*, 4*S*, 5*S*, and 7*S*.

Chermebilaene B (**2**) was isolated as a colorless, amorphous solid with a determined molecular formula of C_25_H_36_O_9_ (eight degrees of unsaturation) on the basis of (+)-HRESIMS (high resolution electrospray ionization mass spectroscopy) data. The ^1^H and ^13^C NMR spectroscopic data (Table 1) contained 25 carbon signals, which were sorted by DEPT and HSQC experiments into the following categories: six methyls, five methylenes (with one oxygenated), six methines (with two oxygenated), and eight quaternary carbons (with two carbonyl and four oxygenated), as well as two exchangeable protons. Detailed analysis of the ^1^H and ^13^C NMR spectroscopic data revealed that **2** was a spiromeroterpenoid derivative, similar to asnovolin G isolated from the fungus *Aspergillus novofumigatus* [14]. However, one of the methylenes resonating at *δ*_C/H_ 28.4/1.61 (CH_2_-7) in asnovolin G was replaced by an oxymethine unit in **2**, as shown by the HRESIMS data as well as by the observation of additional resonance signals at *δ*_C_ 67.0/*δ*_H_ 3.71 (CH-7) and *δ*_H_ 4.54 (7-OH) in the NMR spectra of **2** (Table 2). The deduction was supported by the COSY and HMBC correlations (Figure 2).

The relative configuration was determined by the detailed analysis of the NOESY data (Figure 3). Key NOESY correlations from H-13*α* to H_3_-15 and H_3_-12, and from the protons of 7-OH to H_3_-12, revealed the cofacial orientation of these groups, while the NOE cross-peaks from H-5 to H-1*α* and H-7 placed these groups on the opposite face, which determined the relative configuration of the A/B rings. Furthermore, the NOE correlations from H-3’ to H_3_-8’ and H-4’, and from H-5’ to H-4’ and H-11*β*, indicated the cofacial orientation of these groups, while correlations from H_3_-9’ to H_3_-10’ placed the two methyls on the opposite face. The (5R*, 7S*, 8S*, 9S*, 10S*, 1’S*, 2’S*, 3’S*, 4’R*, 5’S*, 6’R*) relative configuration could thus be deduced for **2**.

**Figure 5:**
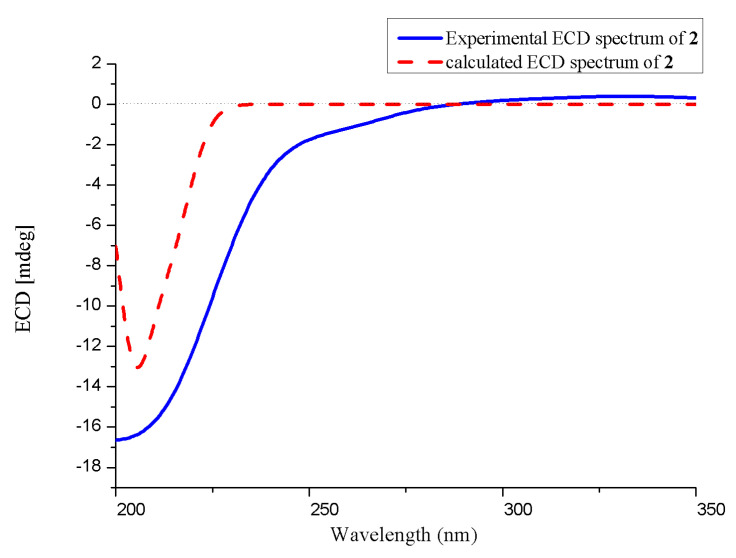
Experimental and calculated ECD (electronic circular dichroism) spectra of **2**.

In order to determine the absolute configuration, a conformational search, DFT (density functional theory) optimizations, and TDDFT-ECD (Time-dependent density functional theory electronic circular dichroism) calculations using density functional theory (B3LYP/6-31G(d)) in Gaussian 09 [15] were performed on the arbitrarily chosen (5R*, 7S*, 8S*, 9S*, 10S*, 1’S*, 2’S*, 3’S*, 4’R*, 5’S*, 6’R*)-enantiomer of **2**. The ECD spectra that were computed for this enantiomer at various levels reproduced well the experimental ECD spectrum, allowing the elucidation of the absolute configuration as 5*R*, 7*S*, 8*S*, 9*S*, 10*S*, 1’*S*, 2’*S*, 3’*S*, 4’*R*, 5’*S*, 6’*R* (Figure 5). 

In addition to compounds 1 and 2, three known sesquiterpenoids, namely sesquicaranoic acid B (3) [16], cyclonerodiol (4) [17], and bisabol-l-on-13-säuremethylester (5) [18], were isolated and identified from the co-culture extract. Neither fungus produced these compounds when cultured alone under the same conditions. 

### 2.2. Biological Activities of the Isolated Compounds

The isolated compounds, as well as the hydrolysis products **6** and **7**, were evaluated for antimicrobial activity [19] against nine human- and aqua-pathogenic bacteria, *Aeromonas hydrophilia*, *Edwardsiella ictarda, E. tarda*, *Escherichia coli*, *Staphylococcus aureus*, *Vibrio alginolyticus*, *V. anguillarum*, *V. harveyi*, and *V. parahemolyticus*, as well as four plant-pathogenic fungi, *Alternaria solani*, *Ceratobasidium cornigerum*, *Colletotrichum glecosporioides*, and *Fusarium graminearum* (Table 2). Compound **1** exhibited significant activity against *Edwardsiella tarda* and *Ceratobasidium cornigerum*, with MIC values of 0.25 and 0.5 μg/mL, respectively, whereas one of its hydrolysis product 9,12-octadecadienoic acid (**7**) demonstrated activities against each of the tested pathogens, with MIC (minimum inhibitory concentration) values ranging from 1.0 to 8.0 μg/mL. In contrast, compounds **2–6** were inactive toward all pathogens in our experiments. These data indicated that the incorporation of a fatty acid into the acorane-type sesquiterpenoid derivative likely significantly increases the antimicrobial activity. The addition of a fatty acid could improve the penetration into the cell membrane, which may cause the antimicrobial activity [20].

## 3. Materials and Methods 

### 3.1. General

Optical rotations were measured on an Optical Activity AA-55 polarimeter (Optical Activity Ltd., Cambridgeshire, UK). UV spectra were measured on a PuXi TU-1810 UV-visible spectrophotometer (Shanghai Lengguang Technology Co. Ltd., Shanghai, China). ECD spectra were acquired on a JASCO J-715 spectropolarimeter (JASCO, Tokyo, Japan). Quantum chemical calculations were conducted using Gaussian 09 software. NMR spectra were recorded on a Bruker Avance 500 spectrometer (Bruker Biospin Group, Karlsruhe, Germany). Mass spectra were determined on a VG Autospec 3000 or an API QSTAR Pulsar 1 mass spectrometer (VG Instruments, London, UK). HPLC was performed using a Dionex HPLC system (Dionex, Sunnyvale, CA, USA) equipped with a P680 pump, an ASI-100 automated sample injector, and a UVD340U multiple wavelength detector controlled by Chromeleon software (version 6.80). Commercially available Si gel (200-300 mesh, Qingdao Haiyang Chemical Co., Qingdao, China), Lobar LiChroprep RP–18 (40–63 μm, Merck, Darmstadt, Germany), and Sephadex LH–20 (18–110 μm, Merck, Darmstadt, Germany) were used for open column chromatography. Solvents for extraction and purification were distilled prior to use. 

### 3.2. Fungal Material 

The fungus *Penicillium bilaiae* MA-267 was isolated from the rhizosphere of the marine mangrove plant *Lumnitzera racemosa* that was collected at Hainan Island, P. R. China, in March 2013. *P. chermesinum* EN-480 was isolated from the fresh tissue of marine red algal *Pterocladiella tenuis*, collected from Shandong province, P. R. China, in July 2014. The fungi were identified as *Penicillium bilaiae* and *Penicillium chermesinum*, respectively, by sequence analysis of the ITS (internal transcribed spacer) regions of their 18S rDNA, as described previously [21]. The resulting sequence data obtained were deposited in GenBank (accession nos. KP096311 for *P. bilaiae* MA-267 and KT119566 for *P. chermesinum* EN-480). Both strains EN-480 and MA-267 are preserved at the Key Laboratory of Experimental Marine Biology, Institute of Oceanology of the Chinese Academy of Sciences.

### 3.3. Fermentation

*P. bilaiae* MA-267 and *P. chermesinum* EN-480 were each grown on PDA (potato dextrose agar) medium at 28 °C for four days, and *P. chermesinum* EN-480 was then inoculated into 1 L conical flasks (100 × 100 mL, a total of 10 L), each containing 100 mL of rice medium (70 g rice, 0.3 g peptone, 0.1 g corn syrup, and 100 mL naturally sourced and filtered seawater that was obtained from the Huiquan Gulf of the Yellow Sea near the campus of IOCAS) at room temperature. After 3 days, a full loop of *P. bilaiae* MA-267 was transferred aseptically to each flask culture of *P. chermesinum* EN-480 and re-incubated at room temperature for 28 days.

### 3.4. Extraction and Isolation 

**Scheme 1:**
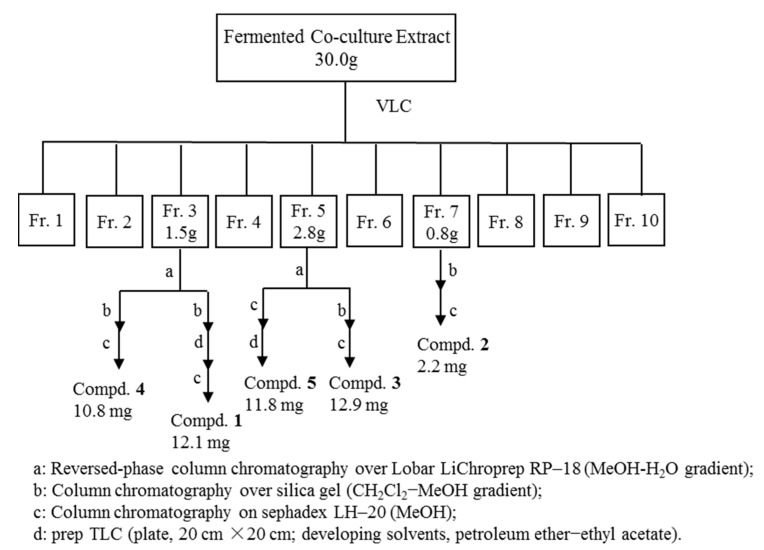
Isolation procedure of compounds **1–5**.

The fermented co-cultures of *P. bilaiae* MA-267 and *P. chermesinum* EN-480 were exhaustively extracted with EtOAc (150 mL/flask). The combined EtOAc solution was concentrated under reduced pressure to give an extract (30 g), which was fractionated by silica gel vacuum liquid chromatography (VLC) using different solvents of increasing polarity from petroleum ether (PE) to MeOH to yield ten fractions (Frs. 1–10), based on TLC (thin layer chromatography) analysis (developing solvents, CH_2_Cl_2_−MeOH, 20:1). Fraction 3 (1.5 g) was further purified by reversed-phase column chromatography (CC) over Lobar LiChroprep RP–18 with a MeOH-H_2_O gradient (from 20:80 to 100:0) to afford five subfractions (Fr. 3-1 to Fr. 3-5). Further purification of Fr. 3-3 by CC over silica gel with a CH_2_Cl_2_−MeOH gradient (from 80:1 to 10:1) and then on Sephadex LH–20 (MeOH) yielded compound **4** (10.8 mg) (Scheme 1). Fr.3-5 was further purified by CC over silica gel with a CH_2_Cl_2_−MeOH gradient (from 50:1 to 5:1), prep TLC (plate, 20 cm ×20 cm; developing solvents, petroleum ether−ethyl acetate, 5:1), and then on Sephadex LH-20 (MeOH) to yield compound **1** (12.1 mg). Fraction 5 (2.8 g) was further purified by reversed-phase column chromatography (CC) over Lobar LiChroprep RP–18 with a MeOH-H_2_O gradient (from 20:80 to 100:0) to afford five subfractions (Fr. 5-1 to Fr. 5-5). Fr. 5-2 was further purified by Sephadex LH–20 (MeOH) and then prep TLC (plate, 20 cm ×20 cm; developing solvents, petroleum ether−ethyl acetate, 2:1) to afford compound **5** (11.8 mg). Fr. 5-3 was further purified by CC over silica gel with a CH_2_Cl_2_−MeOH gradient (from 60:1 to 10:1) and then on Sephadex LH–20 (MeOH) to yield compound **3** (12.9 mg). Further purification of fraction 7 (0.8 g) by CC over silica gel with a CH_2_Cl_2_−MeOH gradient (from 50:1 to 1:1) and then on Sephadex LH–20 (MeOH) yielded compound **2** (2.2 mg). Sesquiterpenes and spiromeroterpenoid derivatives were the major metabolites of *P. bilaiae* MA-267 and *P. chermesinum* EN-480 when cultured alone, as reported in our previous chemical investigations [9,10,11].

Chermebilaene A (**1**): Colorless oil; (α${)}_{D}^{20}$: −5.25 (*c* 0.05, MeOH); IR *υ*_max_ 2957, 1991, 1734, 1620, 991 cm^−1^; ^1^H and ^13^C NMR data, Table 1; HRESIMS *m/z* 558.4527 (M + NH_4_)^+^ (calcd for C_35_H_60_O_4_N, 558.4517), *m/z* 563.4074 (M + Na)^+^ (calcd for C_35_H_56_O_4_Na, 563.4071).

Chermebilaene B (**2**): Colorless solid; (α${)}_{D}^{20}$: −40.00 (*c* 0.08, MeOH); UV (MeOH) *λ*_max_ (log *ε*) 202 (3.6) nm; IR *υ*_max_ 3549, 3378, 2923, 1803, 1704, 1385, 1320, 1064 cm^−1^; ECD (MeOH) *λ*_max_ (Δε) 209 (−2.7), 259 (−0.28) nm; ^1^H and ^13^C NMR data, Table 1; HRESIMS *m/z* 498.2684 (M + NH_4_)^+^ (calcd for C_25_H_40_O_9_N, 498.2698).

### 3.5. Hydrolysis of Compound 1

Compound **1** (10.0 mg, 18.5 μM) was dissolved in 5 mL of THF/MeOH/H_2_O (2/2/1) mixed solvent, to which an excess amount of lithium hydroxide (10.5 mg, 43.7 μM) was added. The reaction mixture was allowed to stir at room temperature for 36 h and was then evaporated to dryness under reduced pressure. The progress of the reaction was monitored by TLC analysis. The resulting reaction mixture was then dissolved in 2 mL of 10wt% NaH_2_PO_4_, extracted with dichloromethane (3 × 4 mL), dried with Na_2_SO_4_, and then concentrated in vacuo to obtain the product.

### 3.6. Antimicrobial Assay

Antimicrobial assays against two human pathogens (*Escherichia coli*, *Staphylococcus aureus*), seven aquatic bacteria (*Aeromonas hydrophilia*, *Edwardsiella ictarda*, *E. tarda*, *Vibrio alginolyticus*, *V. anguillarum*, *V. harveyi*, and *V. parahemolyticus*), and four plant-pathogenic fungi (*Alternaria solani*, *Ceratobasidium cornigerum*, *Colletotrichum glecosporioides*, and *Fusarium graminearum*), was carried out by the 96-well microtiter plates assay [19]. The pathogens were obtained from the Institute of Oceanology, Chinese Academy of Sciences. Chloramphenicol and amphotericin B were used as positive controls for bacteria and fungi, respectively.

## 4. Conclusions

In summary, we isolated and identified two new meroterpenoid derivatives (**1** and **2**), as well as three known sesquiterpenoids (**3**−**5**), from the co-culture broth of two marine-derived fungi, *P. bilaiae* MA-267 and *P. chermesinum* EN-480, whereas neither fungus could produce these compounds when cultured alone. It is noteworthy that compound **1** represents an unprecedented acorane-type sesquiterpene hybridized with an octadecadienoic acid skeleton, and it may prove useful as an antibiotic agent against aquatic or plant pathogens. The fact that co-cultivation in the present study induced the production of new fungal metabolites with improved antimicrobial activity demonstrates the general value of such co-cultivation experiments and encourages future studies.

## Figures and Tables

**Table 1:** ^1^H (500 MHz) and ^13^C NMR (125 MHz) data of compounds **1**–**2**.

No.	1 (Acquired in CDCl_3_)		2 (Acquired in DMSO-*d*_6_)	
	*δ*_H_ (*J* in Hz)	*δ*_C_	*δ*_H_ (*J* in Hz)	*δ*_C_
1	2.74, overlap	57.4, CH	*α* 1.82, ddd (12.7, 9.6, 3.5) *β* 2.71, ddd (12.7, 10.3, 6.3)	25.8, CH_2_
2	5.21, td (8.6, 4.2)	77.8, CH	2.33, m	29.3, CH_2_
3	*α* 2.53, dt (14.7, 8.6) *β* 2.02, overlap	38.7, CH_2_		173.2, C
4	2.02, overlap	38.3, CH		73.0, C
5		46.9, C	1.33, dd (12.6, 2.3)	49.0, CH
6	*α* 1.66, overlap *β* 2.02, overlap	31.9, CH_2_	1.44, m	29.1, CH_2_
7	5.38, m	71.3, CH	3.71, dq (10.0, 4.0)	67.0, CH
8		132.1, C	2.39, m	46.6, CH
9	5.49, dd (3.3, 1.8)	125.3, CH		92.1, C
10	*α* 1.70, dd (13.1, 3.3) *β* 1.98, dd (13.1, 1.8)	34.8, CH_2_		44.7, C
11		142.8, C	*α* 2.04, d (13.9) *β* 1.93, d (13.9)	49.7, CH_2_
12	*Z* 4.69, s *E* 4.98, brs	114.4, CH_2_	0.90, d (7.2)	10.2, CH_3_
13	1.76, s	24.4, CH_3_	*α* 4.47, d (12.2) *β* 4.27, d (12.2)	66.7, CH_2_
14	1.08, d (7.1)	17.6, CH_3_	1.20, s	34.3, CH_3_
15	1.63, s	19.2, CH_3_	1.15, s	25.9, CH_3_
16		171.0, C		
17	2.00, s	21.4, CH_3_		
1’		173.8, C		128.0, C
2’	2.32, t (7.4)	31.7, CH_2_		107.2, C
3’	1.66, overlap	22.9, CH_2_	5.03, d (8.5)	77.5, CH
4’	1.31, overlap	25.8, CH_2_	2.82, dd (8.5, 6.1)	41.3, CH
5’	1.31, overlap	27.4, CH_2_	2.05, m	40.1, CH
6’	1.31, overlap	29.3, CH_2_		46.9, C
7’	1.31, overlap	29.4, CH_2_		175.3, C
8’	2.05, overlap	31.5, CH_2_	1.64, s	22.0, CH_3_
9’	5.40, dd (10.9, 5.5)	130.4, CH	1.06, d (7.2)	11.7, CH_3_
10’	5.34, dd (10.9, 6.5)	128.2, CH	0.88, s	18.6, CH_3_
11’	2.74, overlap	29.8, CH_2_		
12’	5.34, dt (10.9, 6.5)	128.1, CH		
13’	5.40, dd (10.9, 5.5)	130.2, CH		
14’	2.05, overlap	29.5, CH_2_		
15’	1.31, overlap	29.3, CH_2_		
16’	1.31, overlap	25.2, CH_2_		
17’	1.31, overlap	22.7, CH_2_		
18’	0.89, t (6.8)	14.0, CH_3_		
4-OH			4.36, s	
7-OH			4.54, d (4.0)	

**Table 2:** Antimicrobial activities of compounds **1–7** (MIC, μg/mL) ^a^.

Strains	Compounds			
	1	2–6	7	Positive Control
*A. hydrophilia* ^b^	–	–	4.0	0.5
*E. tarda* ^b^	0.25	–	1.0	0.25
*V. anguillarum* ^b^	–	–	8.0	0.5
*V. harveyi* ^b^	–	–	4.0	2.0
*V. parahemolyticus* ^b^	–	–	4.0	1.0
*C. cornigerum* ^c^	0.5	–	8.0	0.5

^a^ (–) = MIC > 32 μg/mL; ^b^ chloramphenicol as positive control; ^c^ amphotericin B as positive control.

## Supplementary Materials

The following are available online at https://www.mdpi.com/1660-3397/18/7/339/s1, Figure S1: HRESI mass spectrum of compound **1**. Figure S2: ^1^H NMR (500 MHz, CDCl_3_) spectrum of compound **1**. Figure S3: ^13^C NMR (125 MHz, CDCl_3_) and DEPT spectra of compound **1**. Figure S4: COSY spectrum of compound **1**. Figure S5: HSQC spectrum of compound **1**. Figure S6: HMBC spectrum of compound **1**. Figure S7: NOESY spectrum of compound **1**. Figure S8: HRESI mass spectrum of compound **1**. Figure S9: ^1^H NMR (500 MHz, DMSO-*d*_6_) spectrum of compound **2**. Figure S10: ^13^C NMR (125 MHz, DMSO-*d*_6_) and DEPT spectra of compound **2**. Figure S11: COSY spectrum of compound **2**. Figure S12: HSQC spectrum of compound **2**. Figure S13: HMBC spectrum of compound **2**. Figure S14: NOESY spectrum of compound **2**. Figure S15: ^1^H NMR (500 MHz, DMSO-*d*_6_) spectrum of compound **6**. Figure S16: ^13^C NMR (125 MHz, DMSO-*d*_6_) and DEPT spectra of compound **6**. Figure S17: COSY spectrum of compound **6**. Figure S18: HMBC spectrum of compound **6**. Figure S19: HMBC spectrum of compound **6**. Figure S20: NOESY spectrum of compound **6**. Figure S21: ^1^H NMR (500 MHz, DMSO-*d*_6_) spectrum of (*S*)-MTPA ester (**6a**). Figure S22: ^1^H NMR (500 MHz, DMSO-*d*_6_) spectrum of (*R*)-MTPA ester (**6b**).
