# Supplementary material for: Chermebilaenes A and B, New Bioactive Meroterpenoids from Co-Cultures of Marine-Derived Isolates of Penicillium bilaiae MA-267 and Penicillium chermesinum EN-480
Source: Mar Drugs. 2020 Jun 28;18(7):339. doi: 10.3390/md18070339 (PMC7401264; doi:10.3390/md18070339)

Supplementary Material

Chermebilaenes A and B, New Bioactive Meroterpenoids from Co-cultures of Marine-derived Isolates of *Penicillium bilaiae* MA-267 and *Penicillium chermesinum* EN-480

Ling-Hong Meng [1,2,*], Xiao-Ming Li [1,2], Hong-Lei Li [1,2], and Bin-Gui Wang [1,2,3,*]

[1] Key Laboratory of Experimental Marine Biology, Institute of Oceanology, Chinese Academy of Sciences, Nanhai Road 7, Qingdao 266071, China; E-Mails: lixmqdio@126.com (X.-M.L.); lihonglei428@126.com (H.-L.L.)

[2] Laboratory of Marine Biology and Biotechnology, Qingdao National Laboratory for Marine Science and Technology, Wenhai Road 1, Qingdao 266237, China

[3] Center for Ocean Mega-Science, Chinese Academy of Sciences, Nanhai Road 7, Qingdao 266071, China

* Correspondence: menglh@ms.qdio.ac.cn (L.-H.M.); Tel: +86-532-8289-8890 (L.-H.M.); wangbg@ms.qdio.ac.cn (B.-G.W.); Tel.: +86-532-8289 8553 (B.-G.W.)

Content

Figure S1. HRESI mass spectrum of compound **1**.

Figure S2. ^{1}H NMR (500 MHz, $CDCl_3$) spectrum of compound **1**.

Figure S3. ^{13}C NMR (125 MHz, $CDCl_3$) and DEPT spectra of compound **1**.

Figure S4. COSY spectrum of compound **1**.

Figure S5. HSQC spectrum of compound **1**.

Figure S6. HMBC spectrum of compound **1**.

Figure S7. NOESY spectrum of compound **1**.

Figure S8. HRESI mass spectrum of compound **2**.

Figure S9. ^{1}H NMR (500 MHz, DMSO-d_6) spectrum of compound **2**.

Figure S10. ^{13}C NMR (125 MHz, DMSO-d_6) and DEPT spectra of compound **2**.

Figure S11. COSY spectrum of compound **2**.

Figure S12. HSQC spectrum of compound **2**.

Figure S13. HMBC spectrum of compound **2**.

Figure S14. NOESY spectrum of compound **2**.

Figure S15. ^{1}H NMR (500 MHz, DMSO-d_6) spectrum of compound **6**.

Figure S16. ^{13}C NMR (125 MHz, DMSO-d_6) and DEPT spectra of compound **6**.

Figure S17. COSY spectrum of compound **6**.

Figure S18. HSQC spectrum of compound **6**.

Figure S19. HMBC spectrum of compound **6**.

Figure S20. NOESY spectrum of compound **6**.

Figure S21. ^{1}H NMR (500 MHz, DMSO-d_6) spectrum of (*S*)-MTPA ester (**6a**).

Figure S22. ^{1}H NMR (500 MHz, DMSO-d_6) spectrum of (*R*)-MTPA ester (**6b**).

Figure S23. HPLC profiles of the EtOAc extract of (a) *P. bilaiae* MA-267, (b) *P. chermesinum* EN-480, and (c) co-culture.

Figure S1. HRESI mass spectrum of compound **1**.

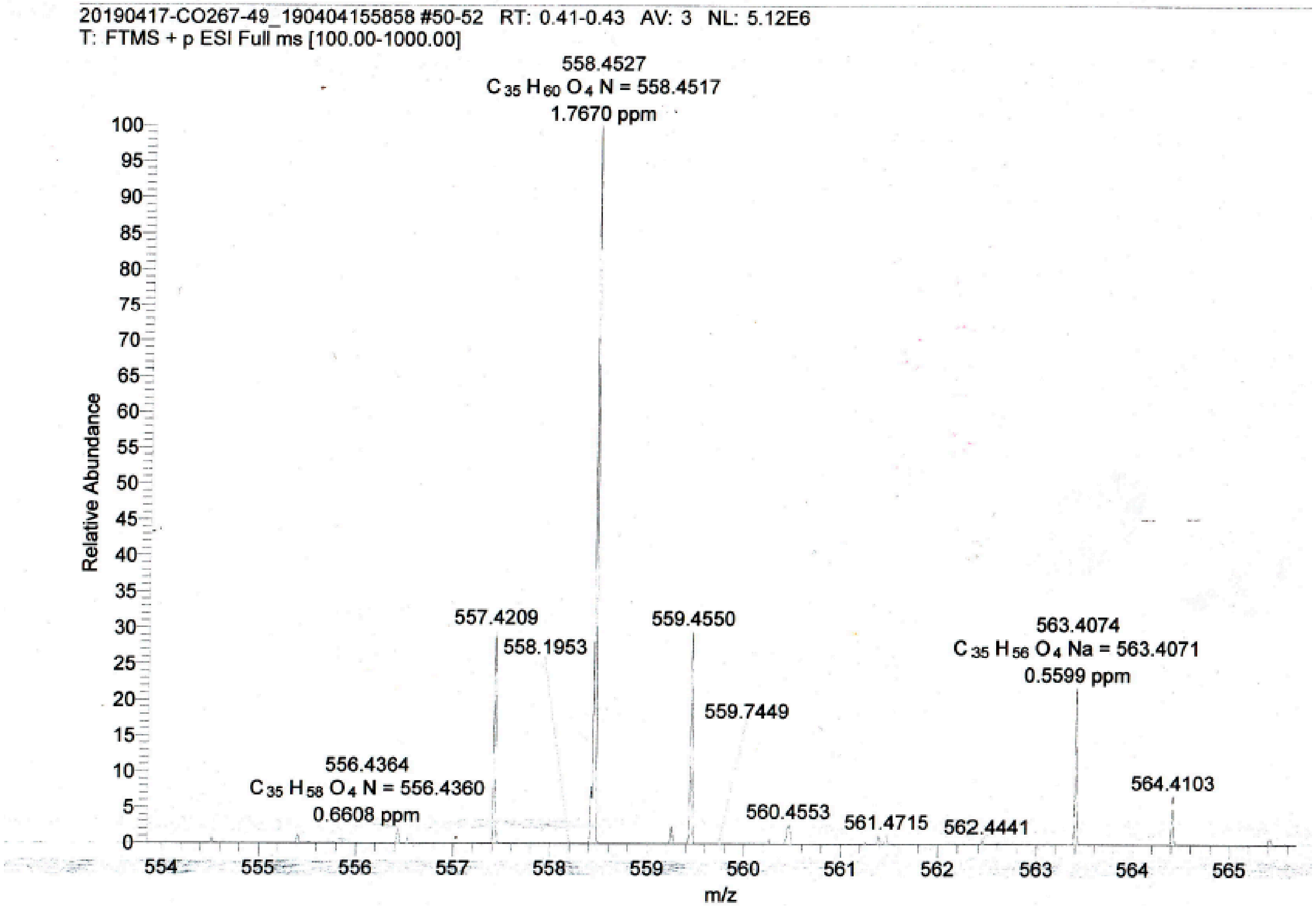

Figure S2. ^{1}H NMR (500 MHz, $CDCl_3$) spectrum of compound **1**.

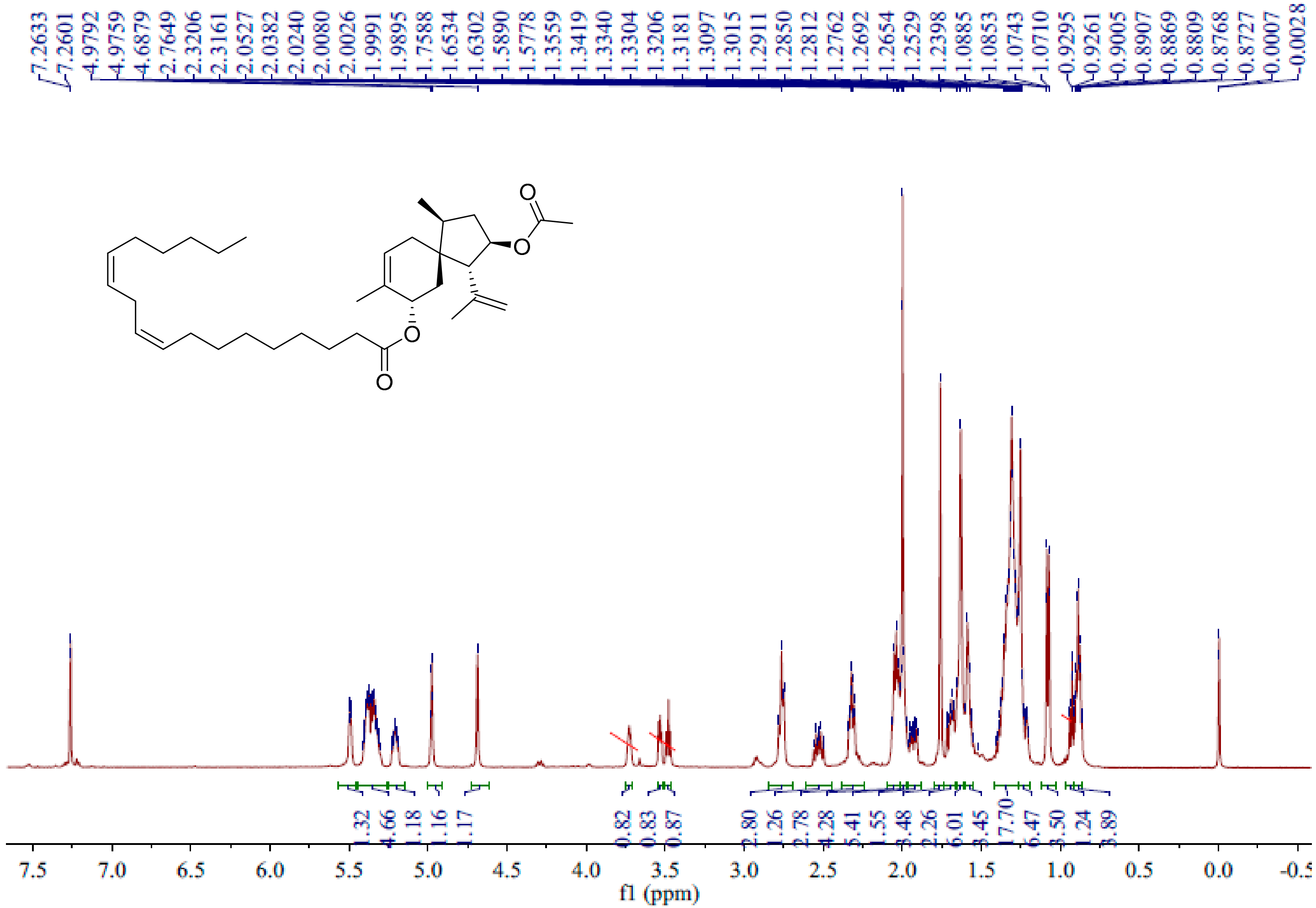

Figure S3. ^{13}C NMR (125 MHz, $CDCl_3$) and DEPT spectra of compound **1**.

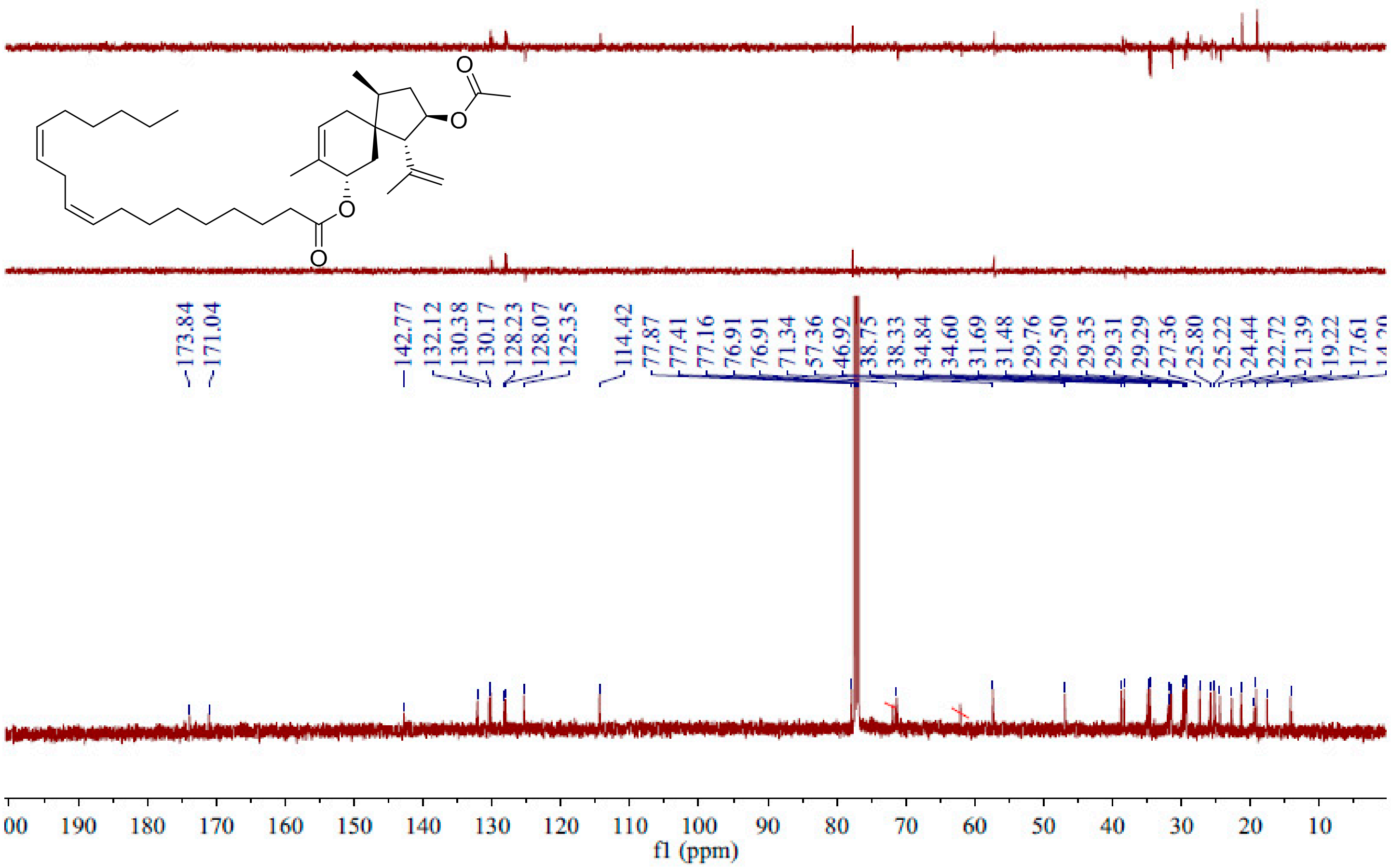

Figure S4. COSY spectrum of compound **1**.

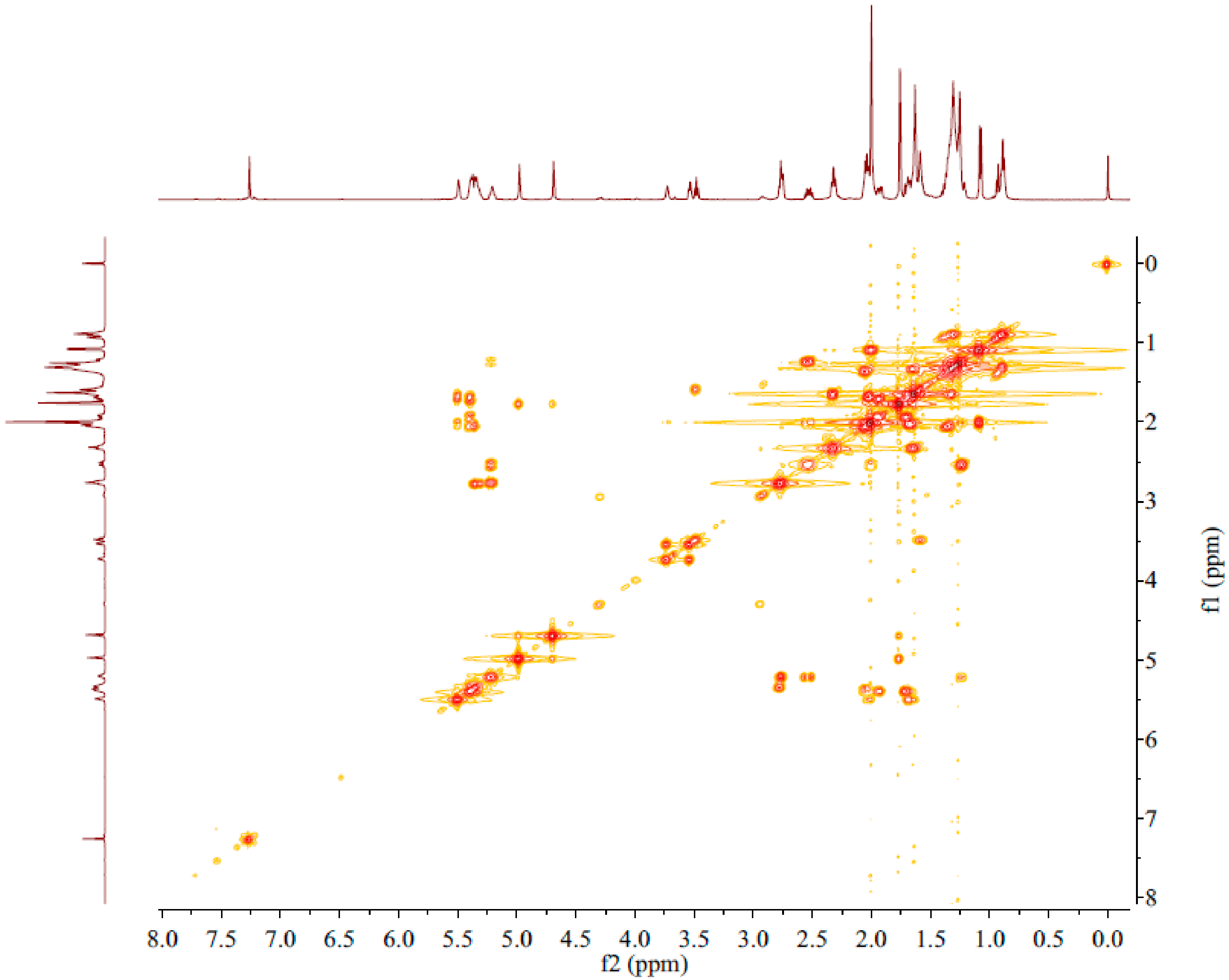

Figure S5. HSQC spectrum of compound **1**.

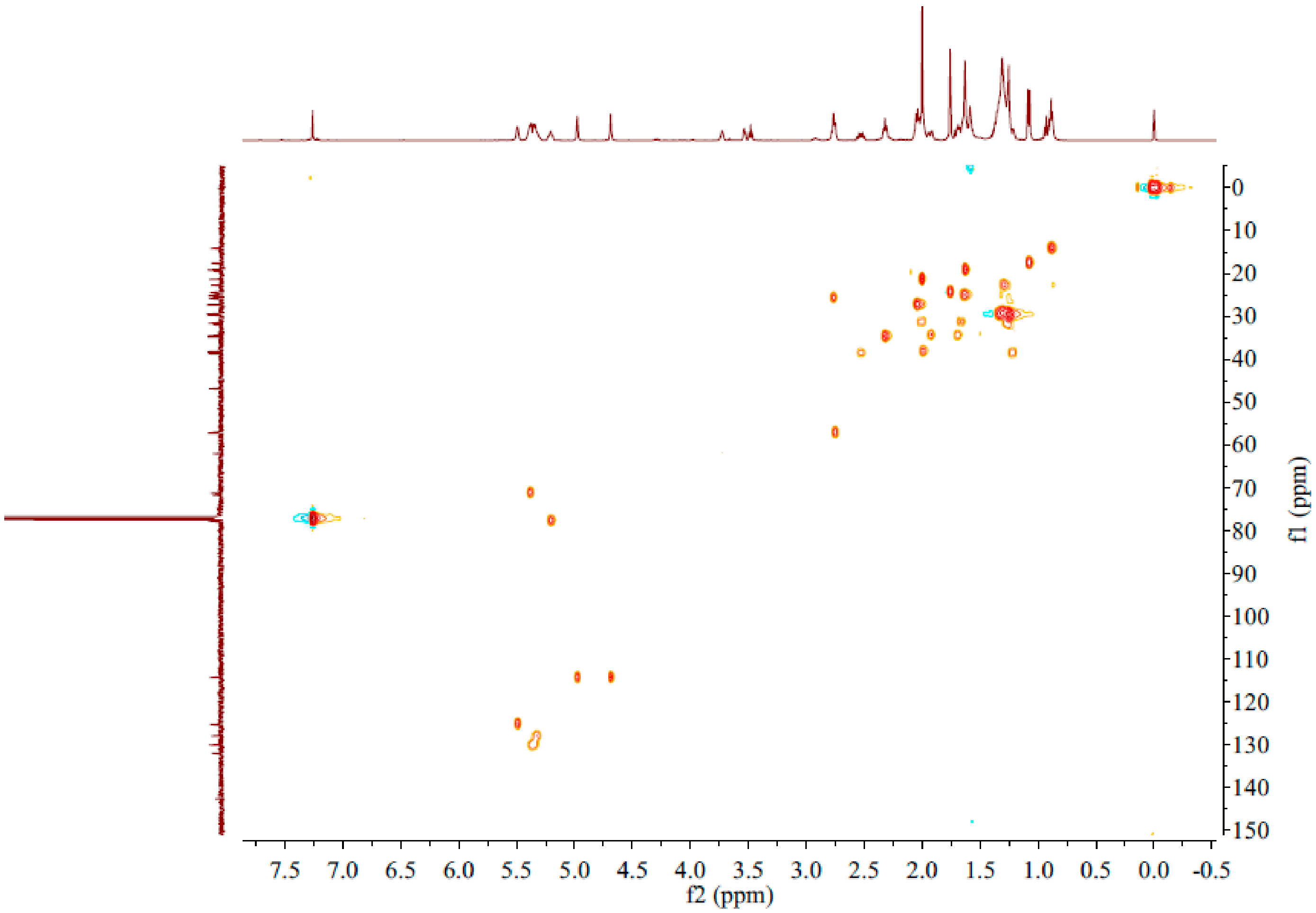

Figure S6. HMBC spectrum of compound **1**.

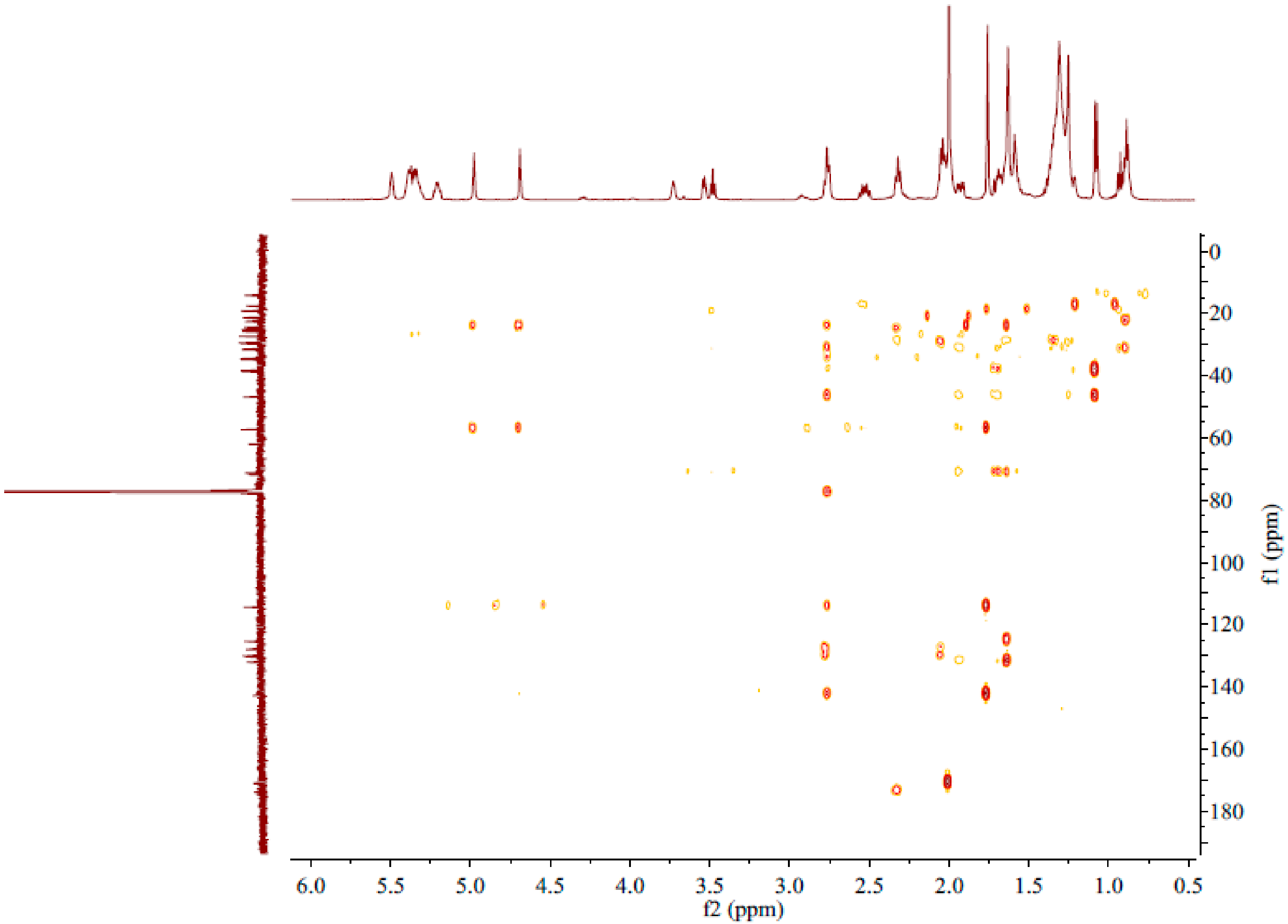

Figure S7. NOESY spectrum of compound **1**.

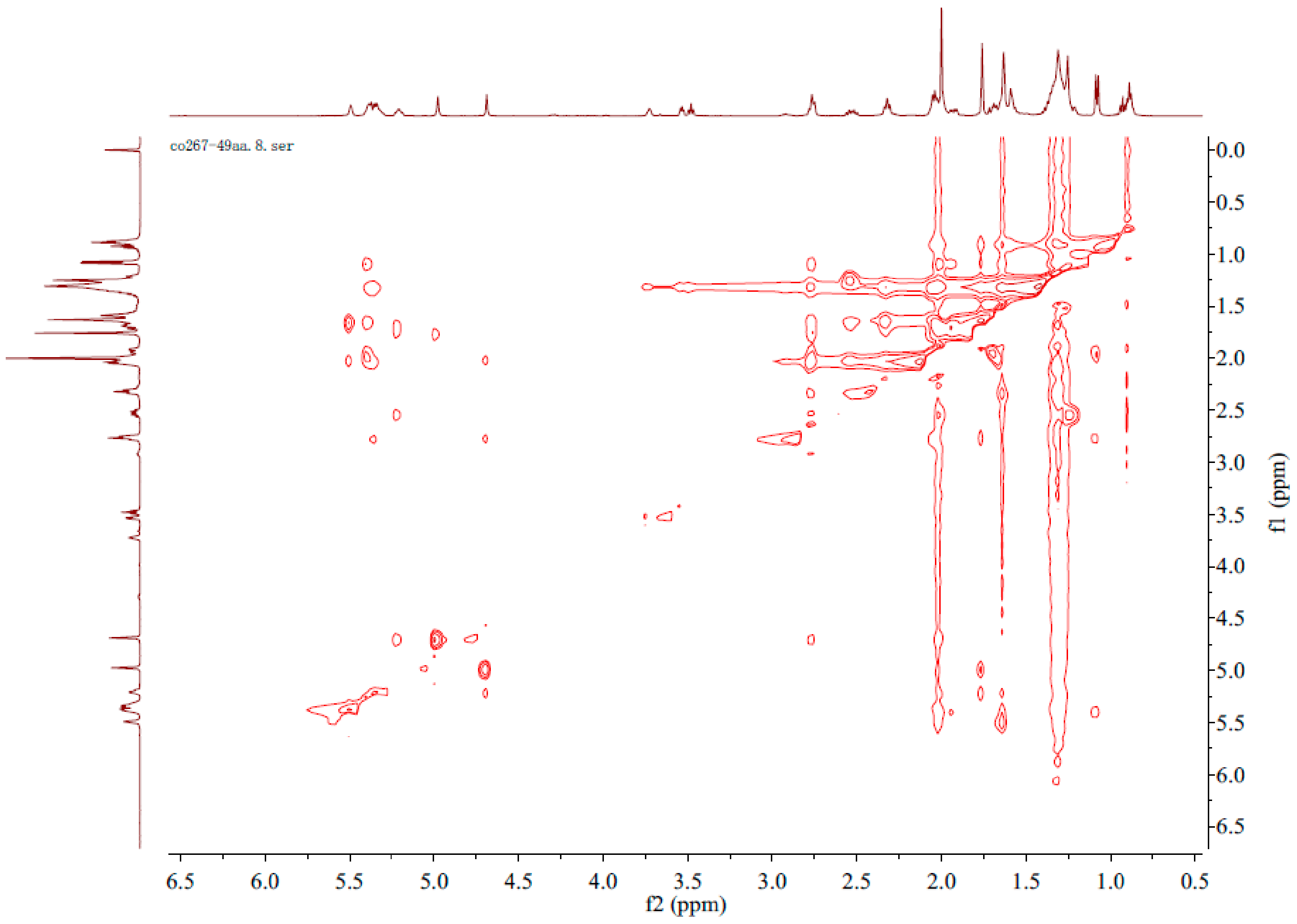

Figure S8. HRESI mass spectrum of compound **2**.

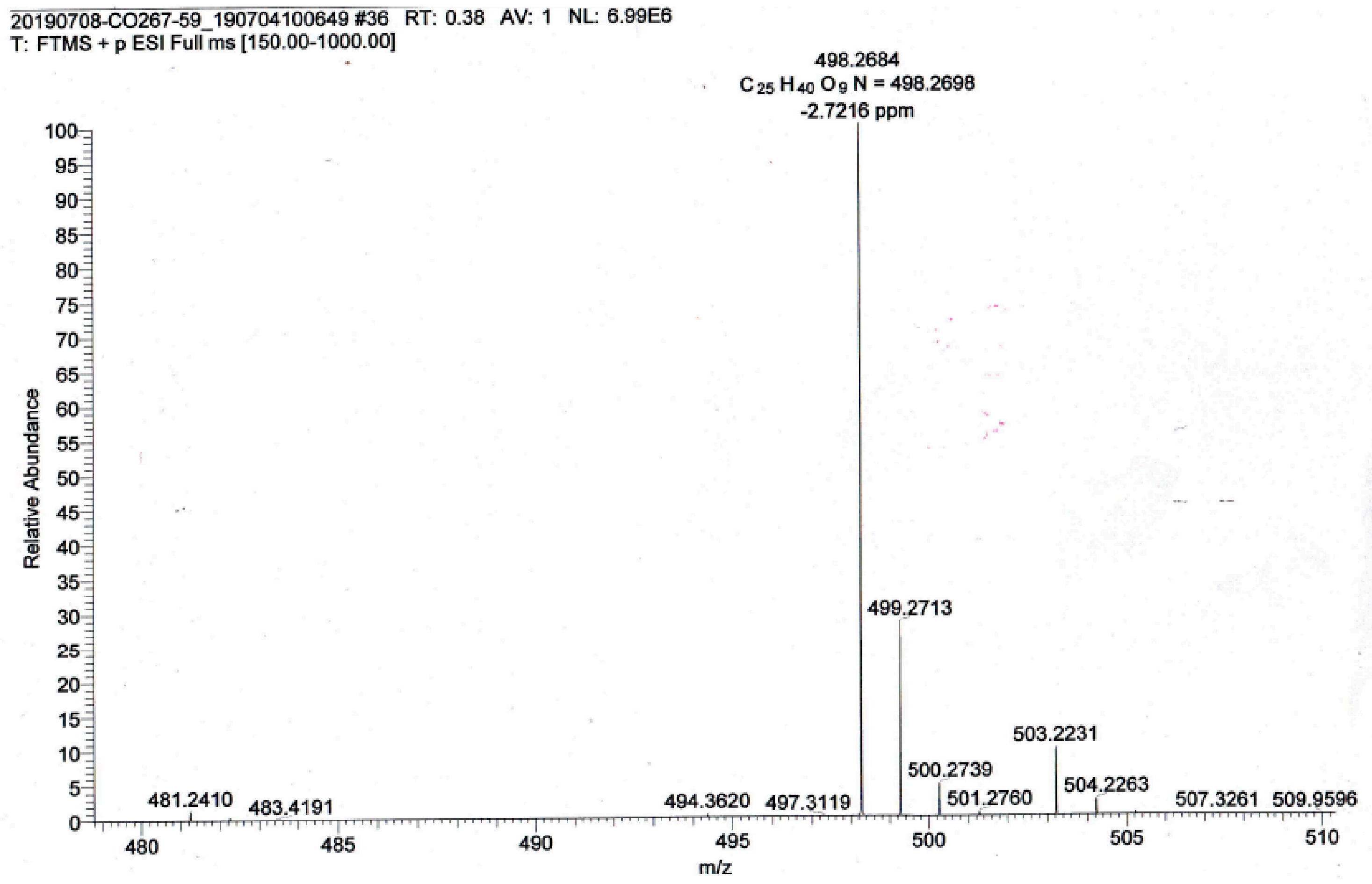

Figure S9. ^{1}H NMR (500 MHz, DMSO-d_6) spectrum of compound **2**.

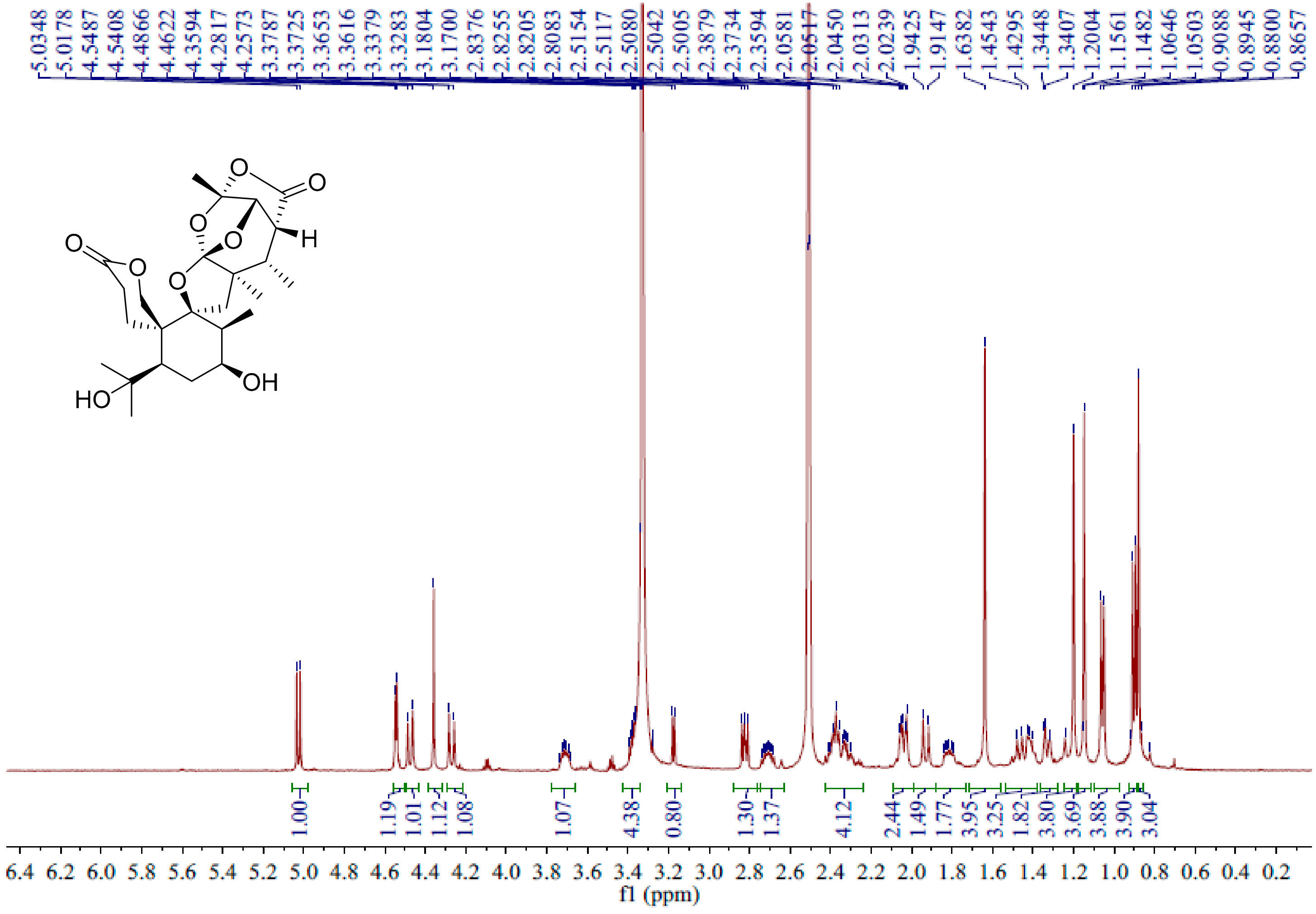

Figure S10. ^{13}C NMR (125 MHz, DMSO-d_6) and DEPT spectra of compound **2**.

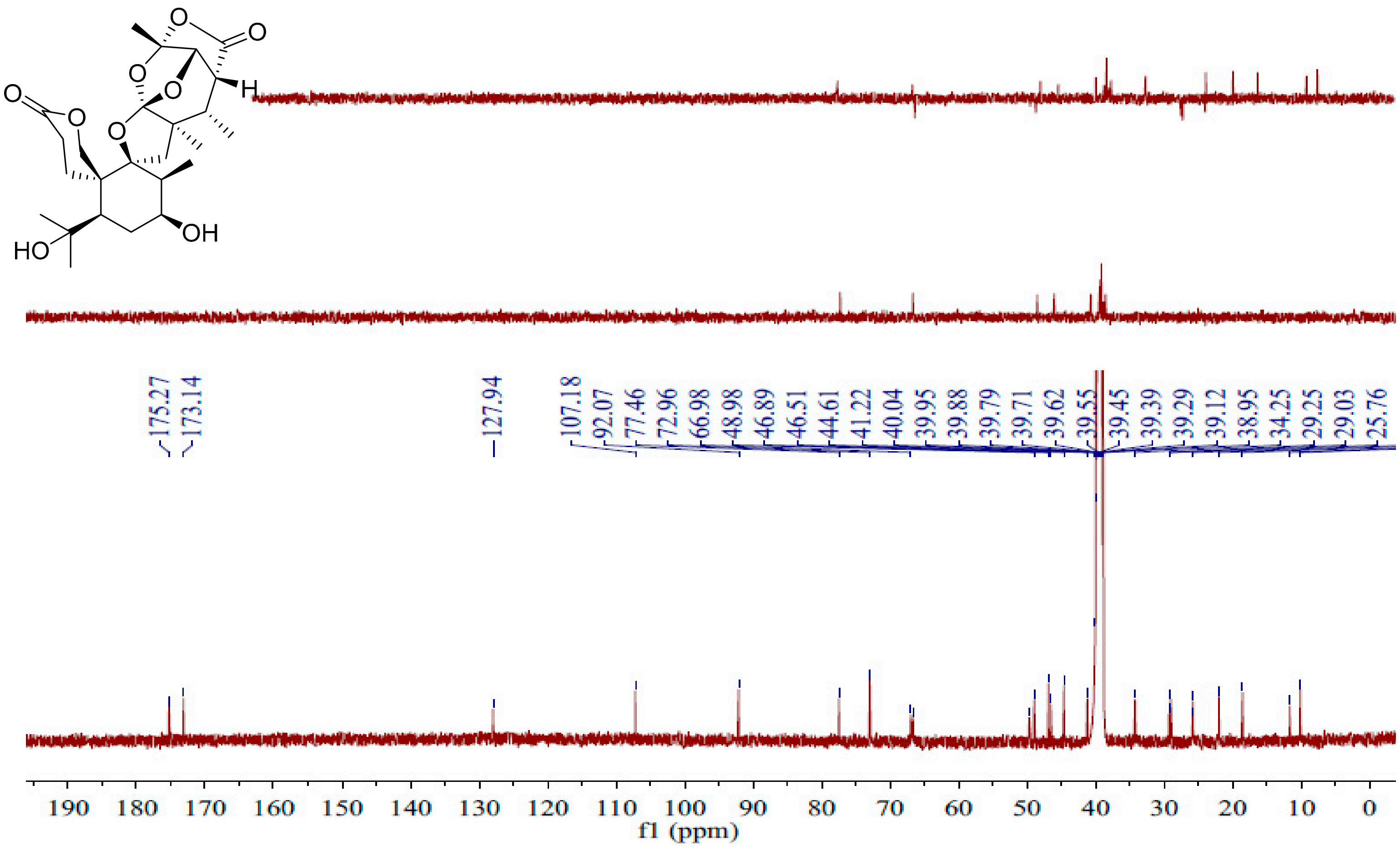

Figure S11. COSY spectrum of compound **2**.

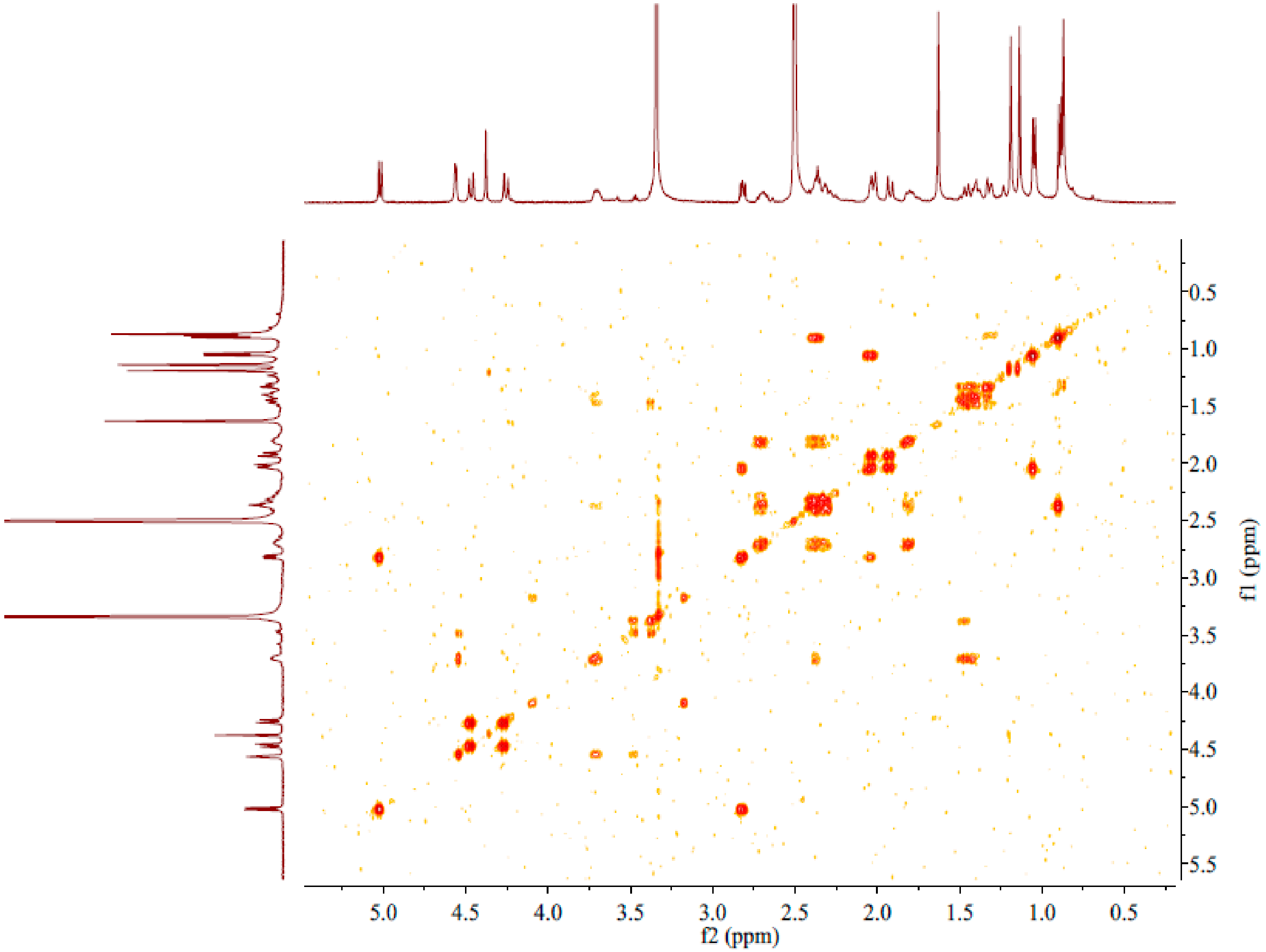

Figure S12. HSQC spectrum of compound **2**.

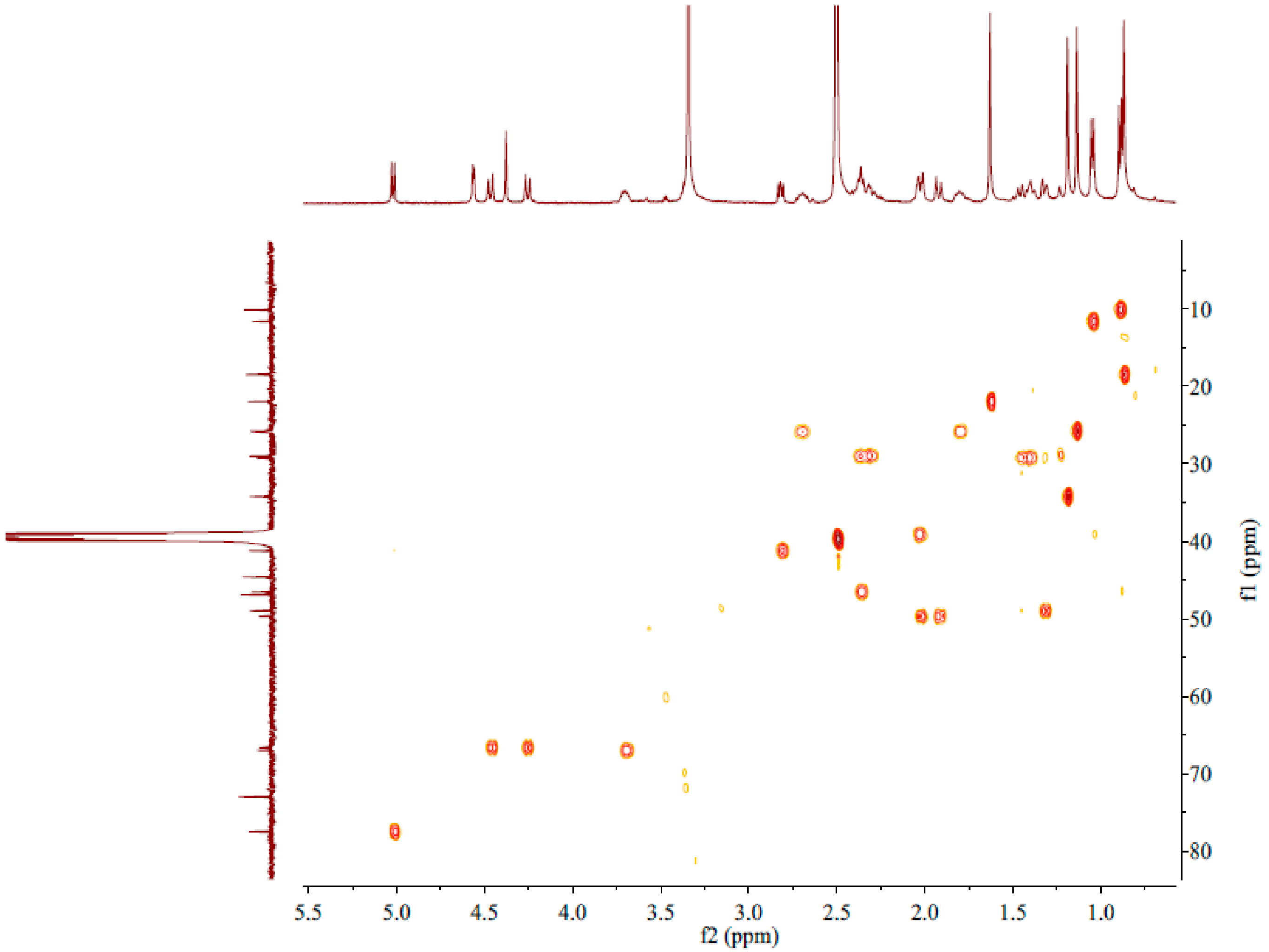

Figure S13. HMBC spectrum of compound **2**.

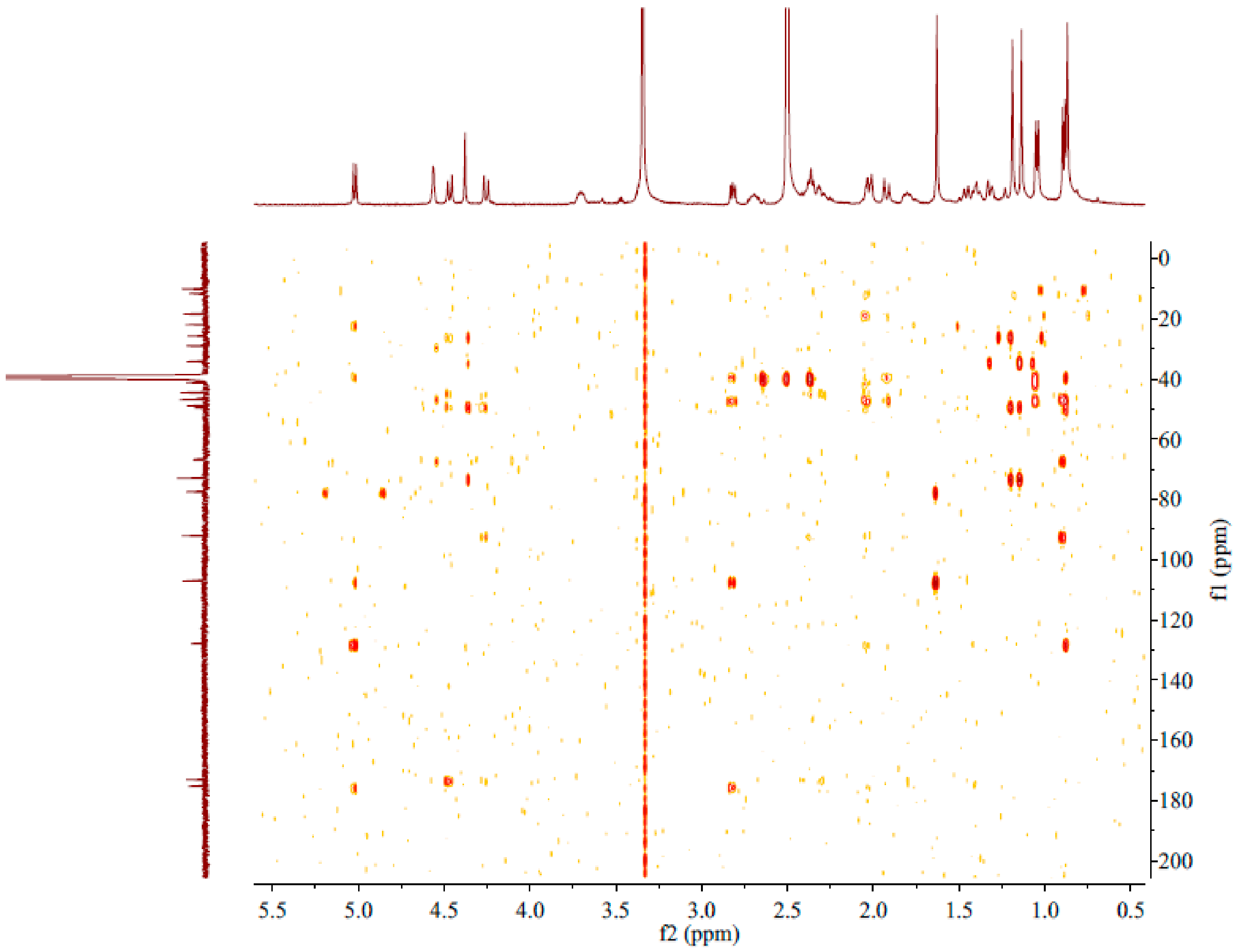

Figure S14. NOESY spectrum of compound **2**.

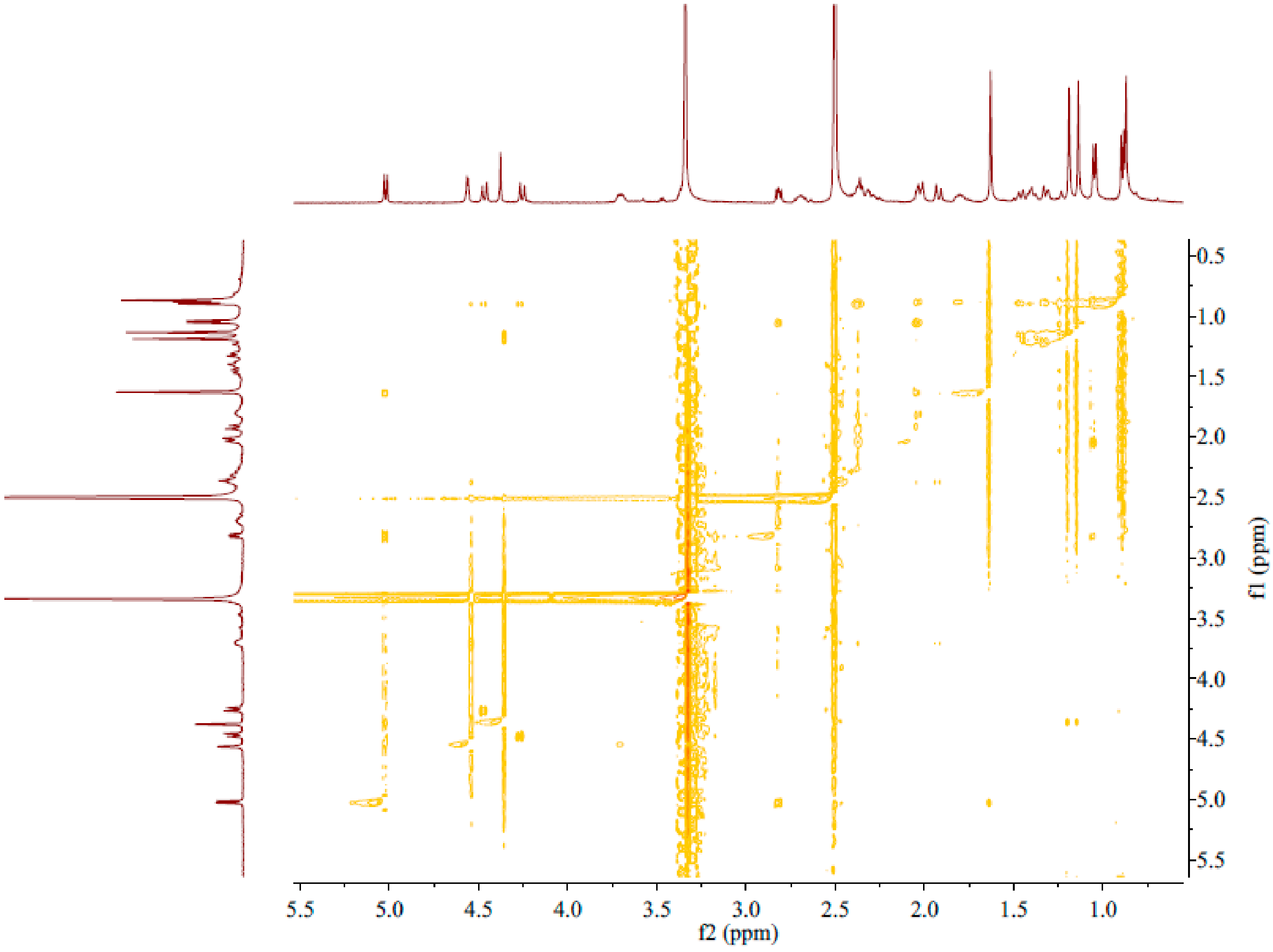

Figure S15. ^{1}H NMR (500 MHz, DMSO-d_6) spectrum of compound **6**.

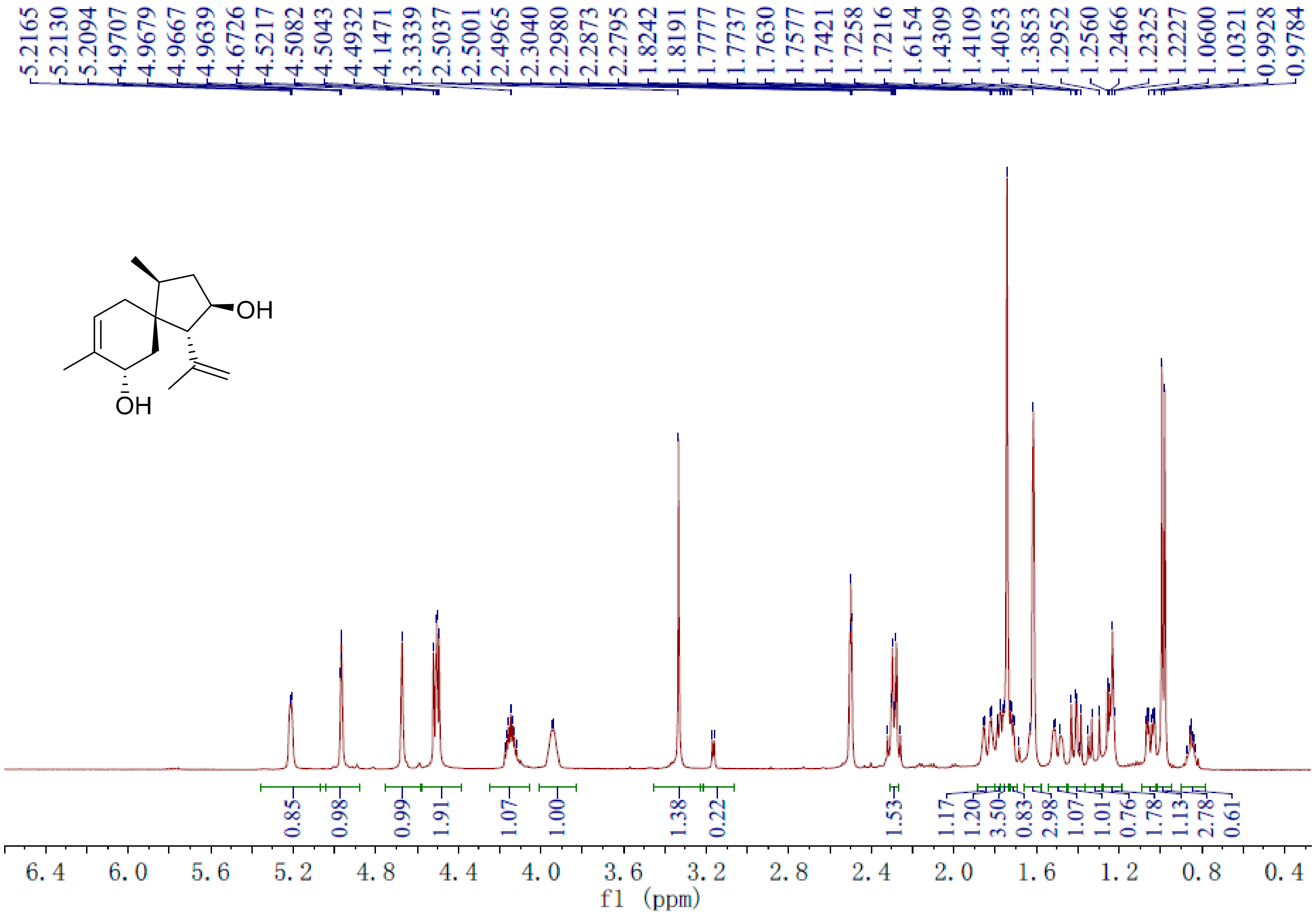

Figure S16. ^{13}C NMR (125 MHz, DMSO-d_6) and DEPT spectra of compound **6**.

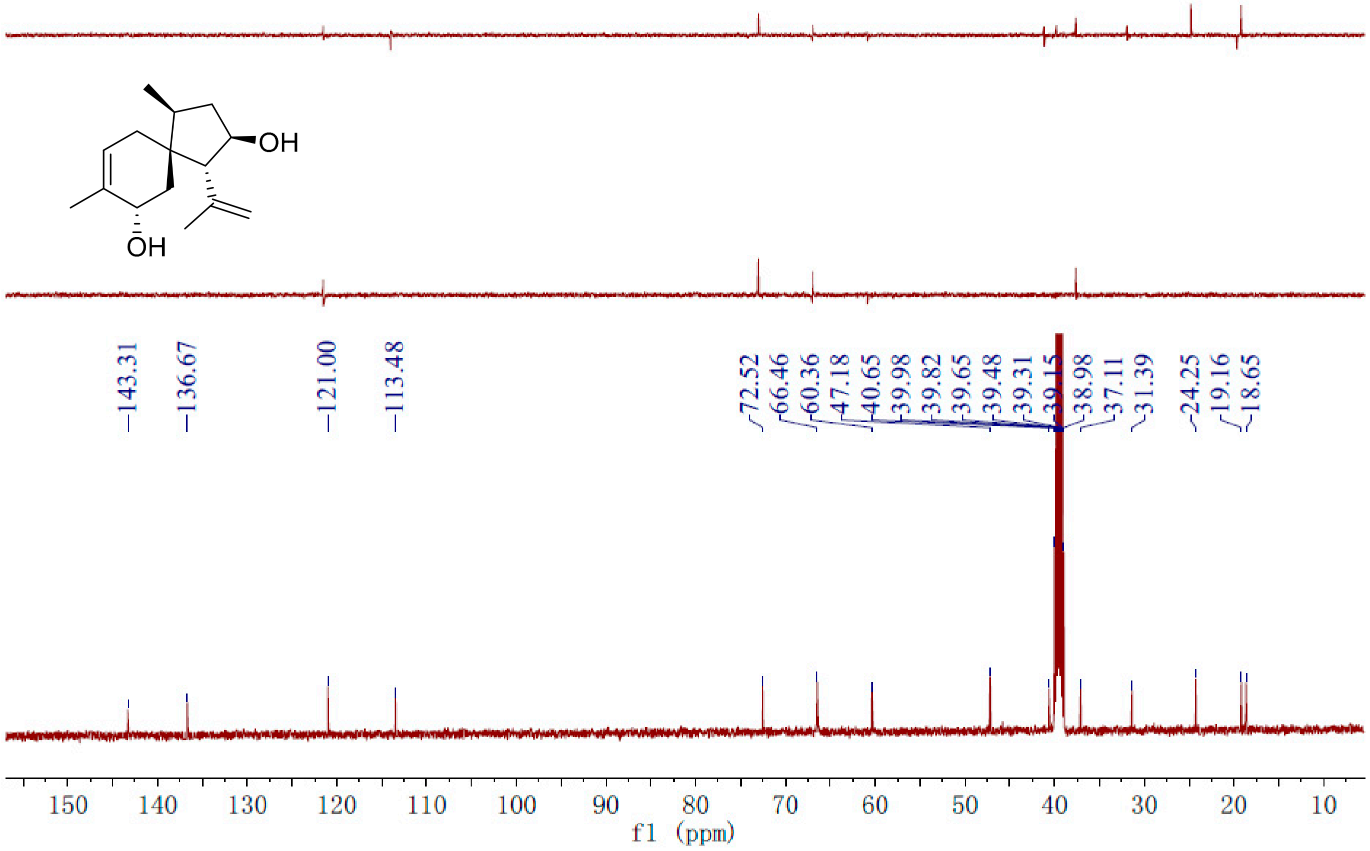

Figure S17. COSY spectrum of compound **6**.

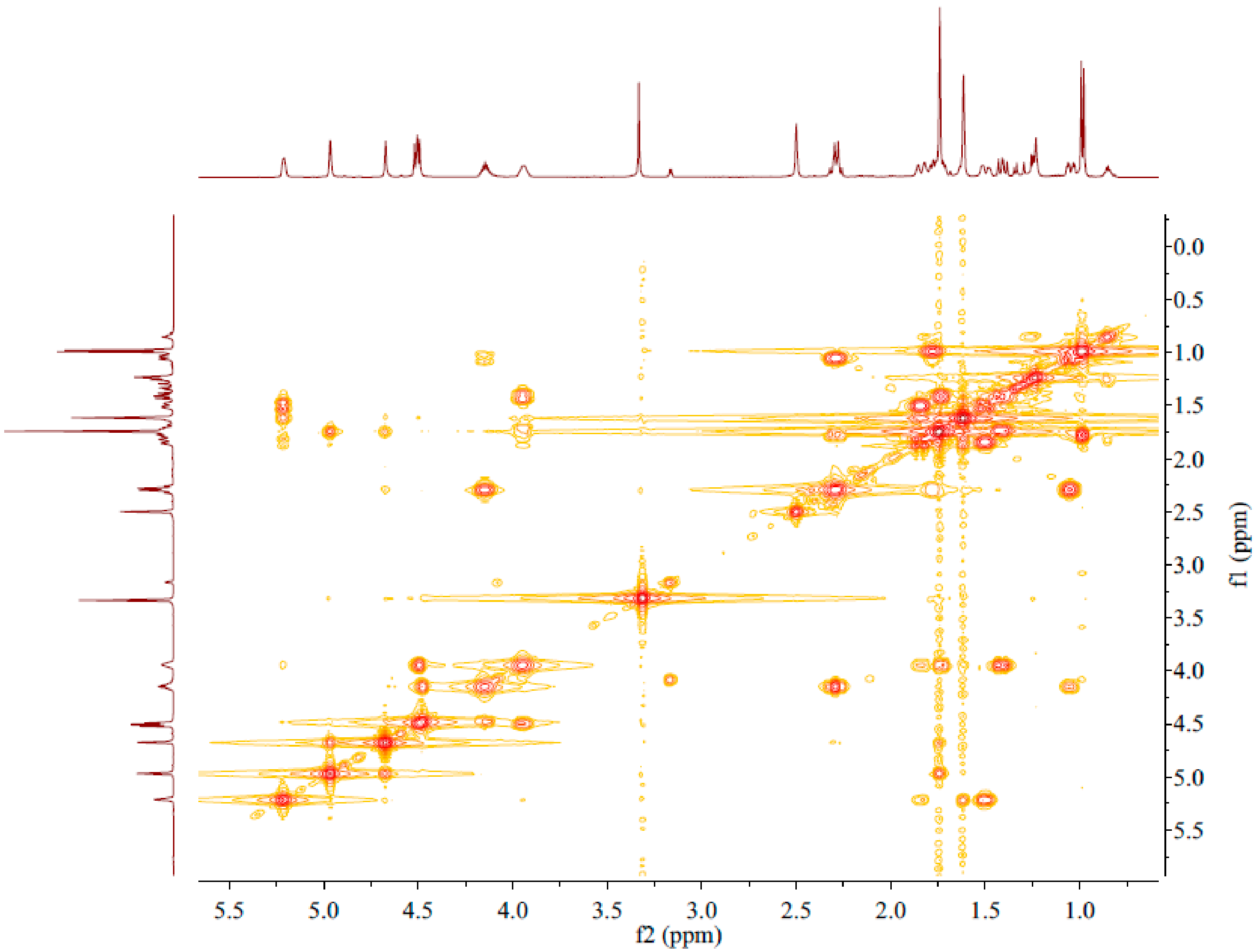

Figure S18. HSQC spectrum of compound **6**.

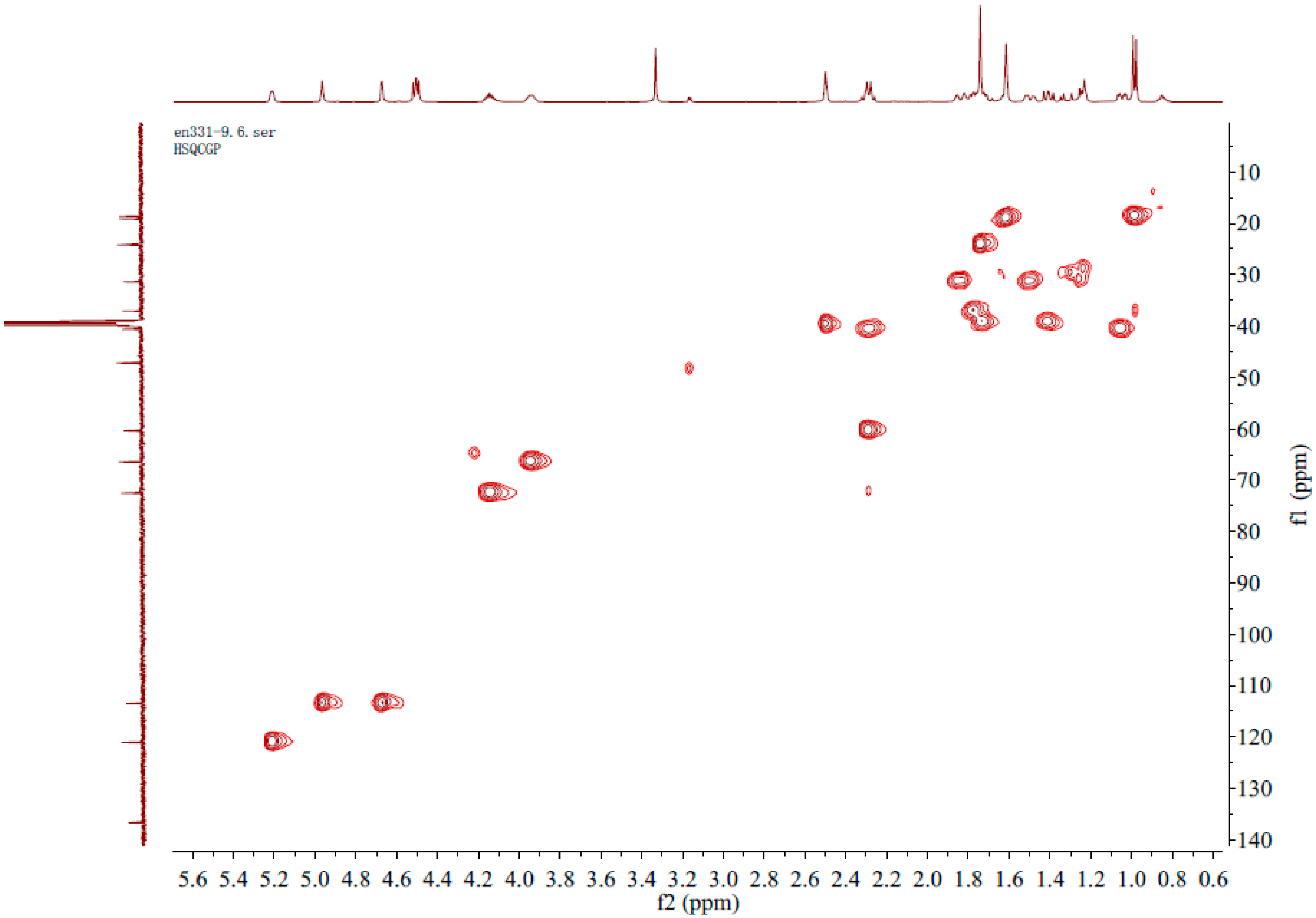

Figure S19. HMBC spectrum of compound **6**.

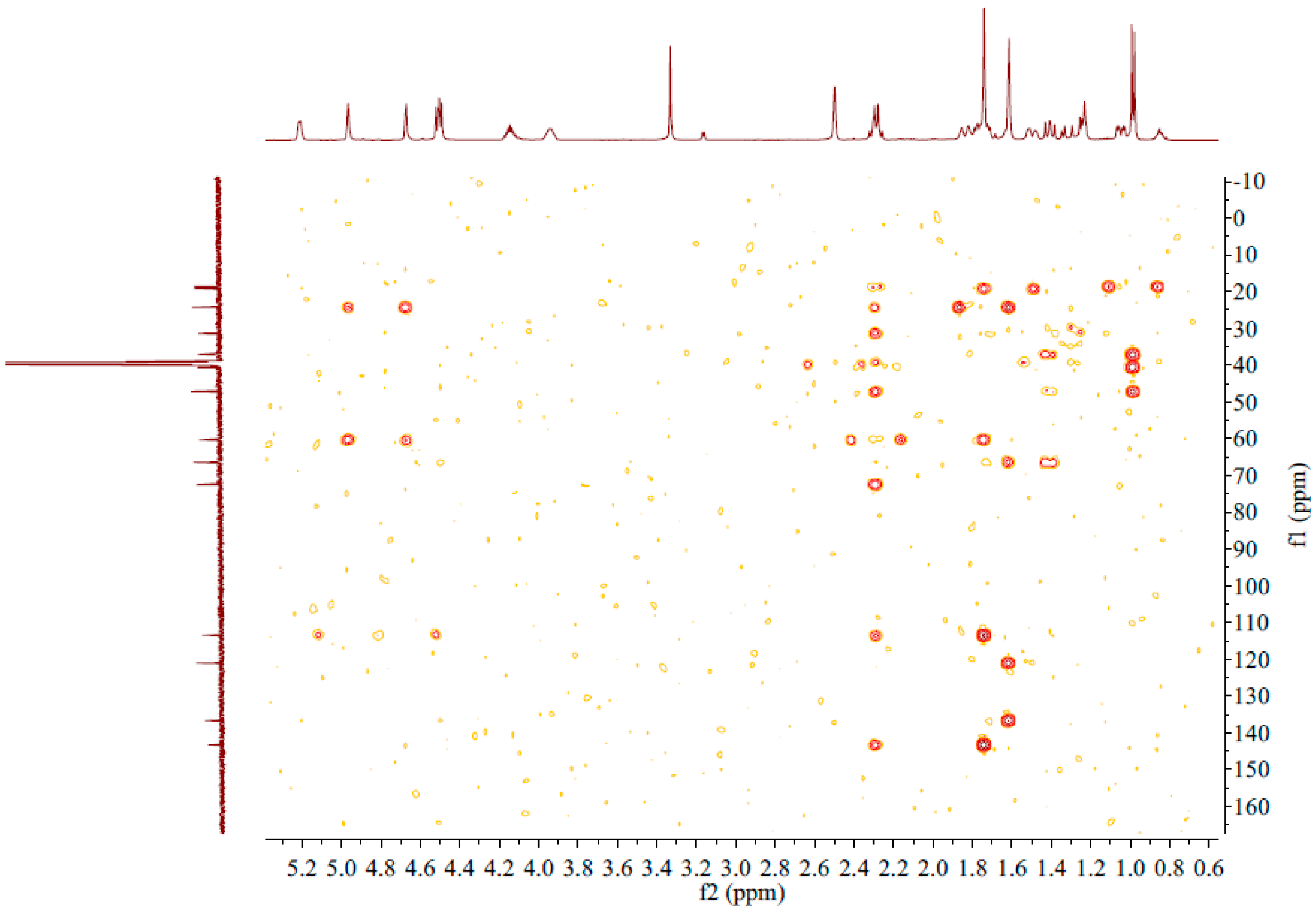

Figure S20. NOESY spectrum of compound **6**.

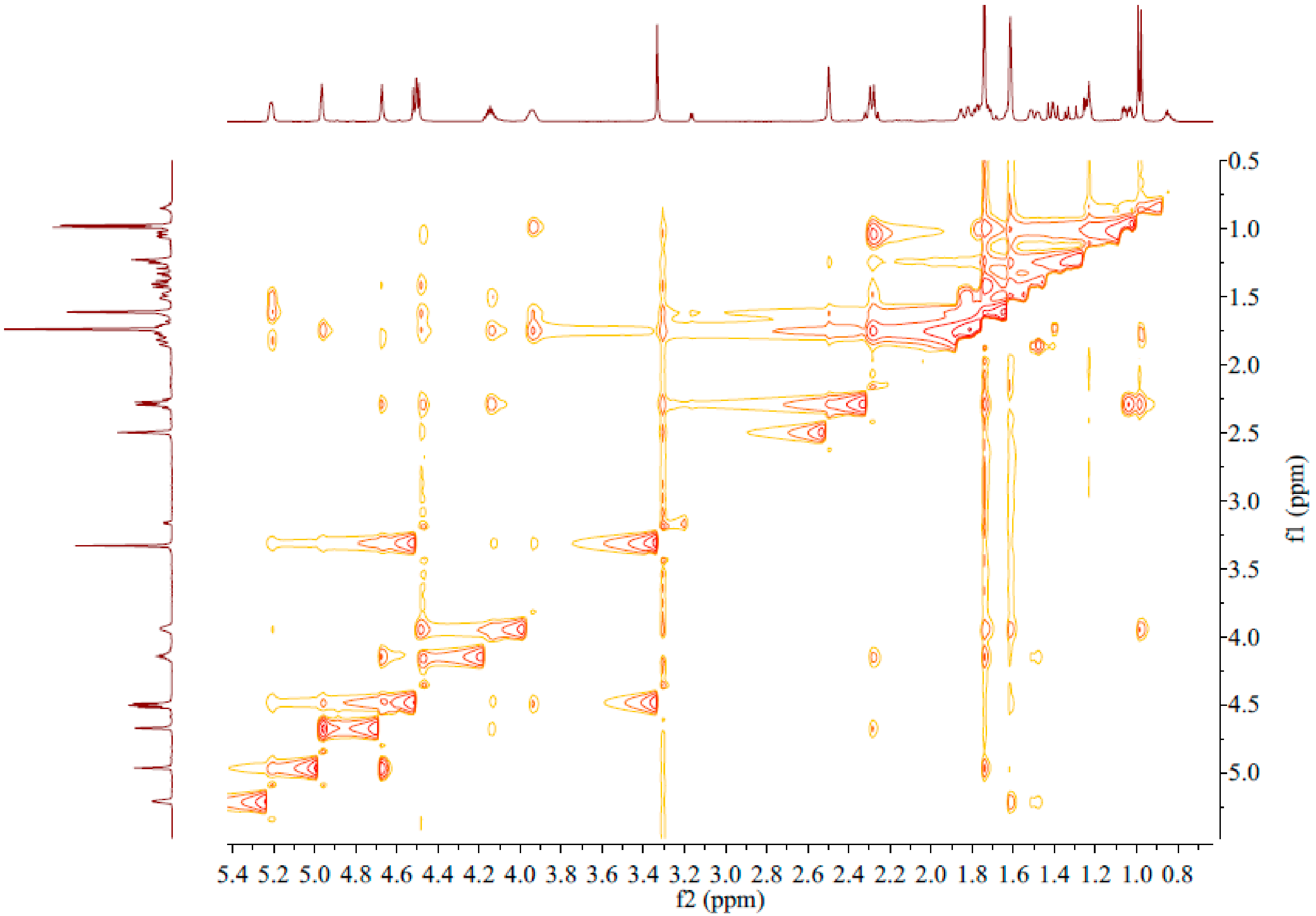

Figure S21. ^{1}H NMR (500 MHz, DMSO-*d*6) spectrum of (*S*)-MTPA ester (**6a**).

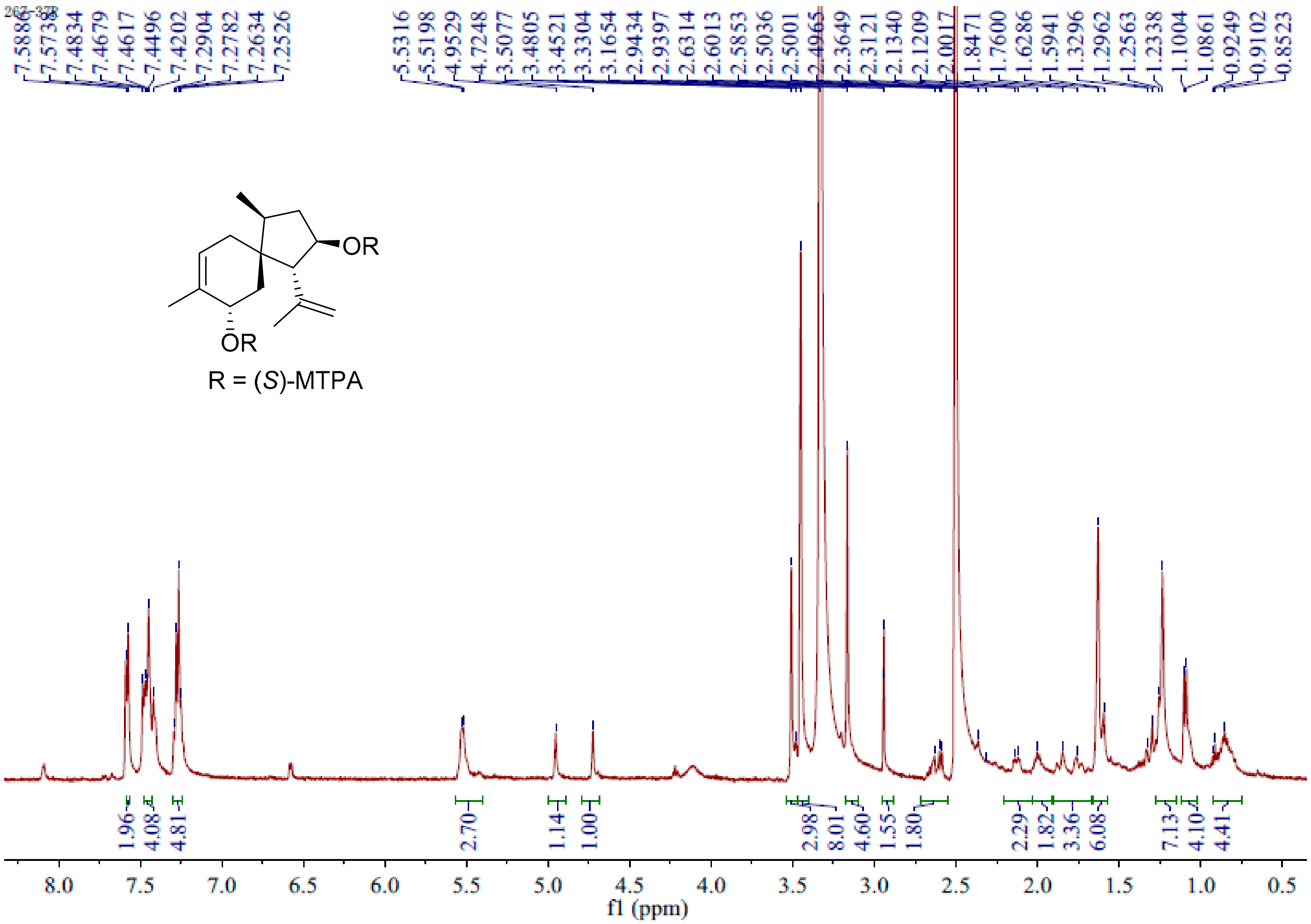

Figure S22. ^{1}H NMR (500 MHz, DMSO-d_6) spectrum of (*R*)-MTPA ester (**6b**).

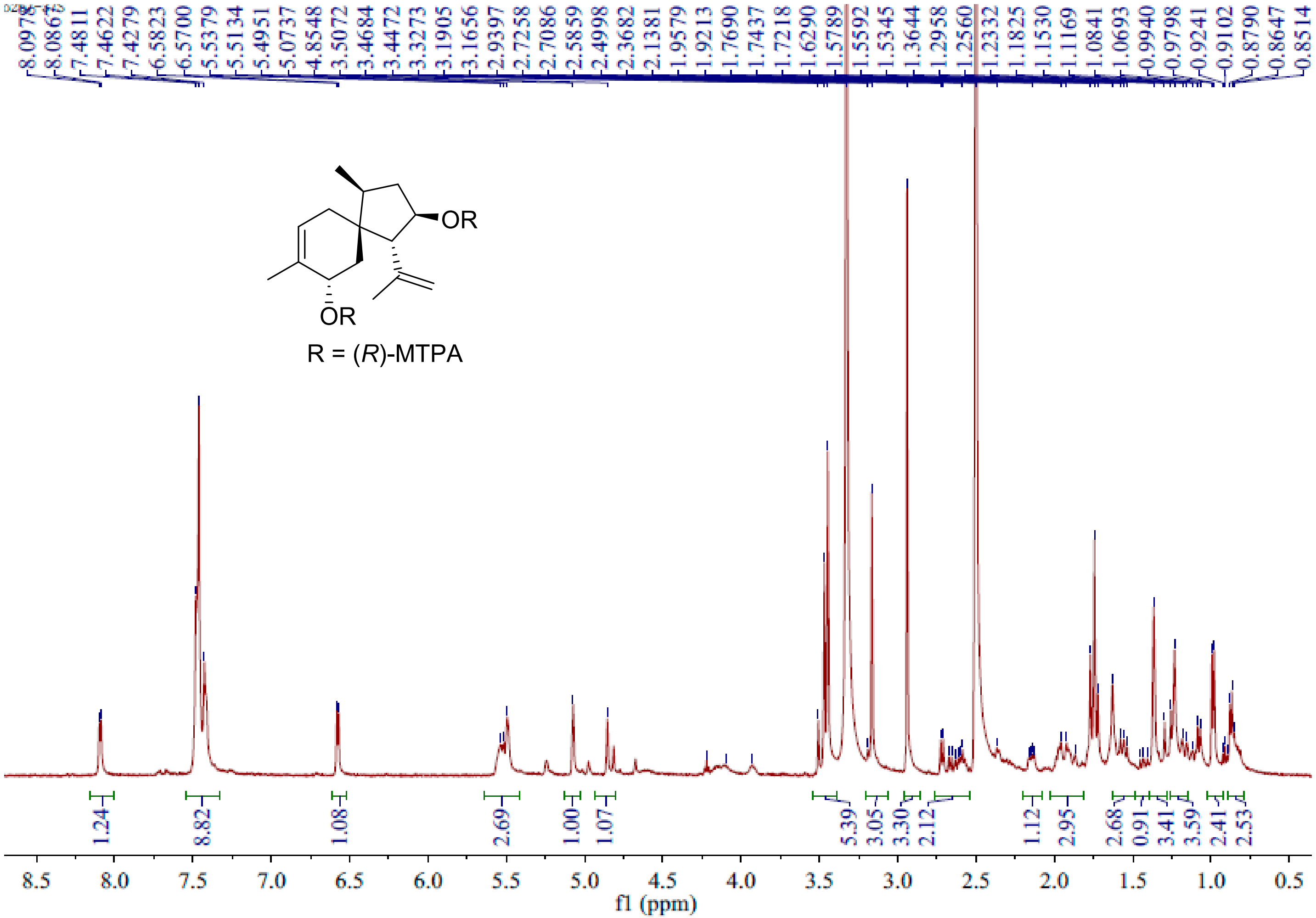

Figure S23. HPLC profiles of the EtOAc extract of (a) *P. bilaiae* MA-267, (b) *P. chermesinum* EN-480, and (c) co-culture.

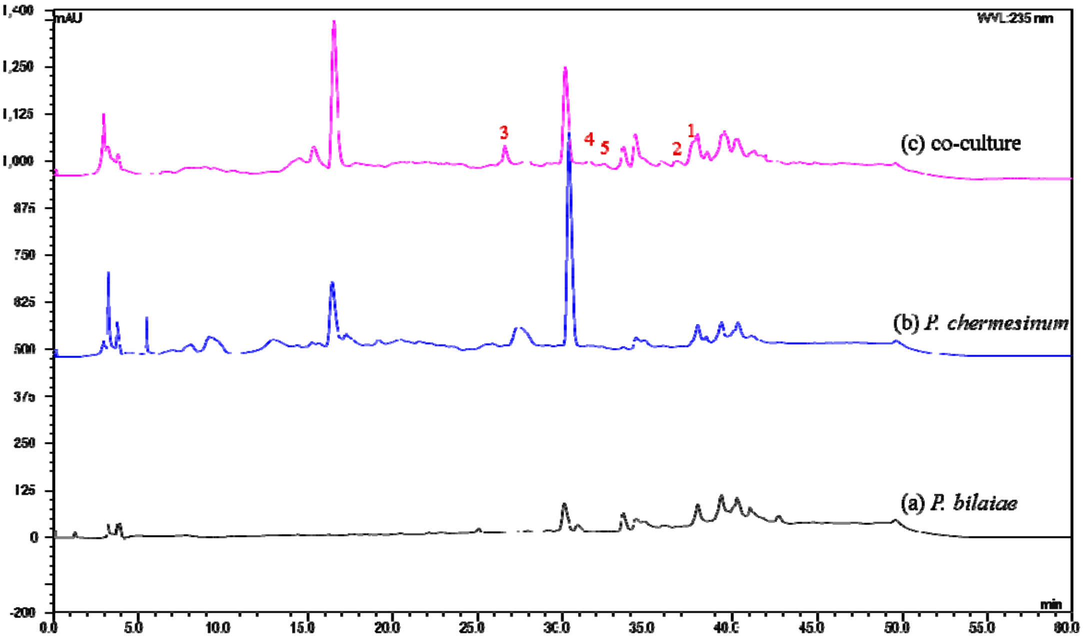

Supplement: Supplementary file 1 [file marinedrugs-18-00339-s001.pdf]
